# Comparative genomics and phylogenomic analyses of lysine riboswitch distributions in bacteria

**DOI:** 10.1371/journal.pone.0184314

**Published:** 2017-09-05

**Authors:** Sumit Mukherjee, Danny Barash, Supratim Sengupta

**Affiliations:** 1 Department of Physical Sciences, Indian Institute of Science Education and Research Kolkata, Mohanpur, India; 2 Department of Computer Science, Ben-Gurion University of the Negev, Beer-Sheva, Israel; Fachhochschule Lubeck, GERMANY

## Abstract

Riboswitches are cis-regulatory elements that regulate the expression of genes involved in biosynthesis or transport of a ligand that binds to them. Among the nearly 40 classes of riboswitches discovered so far, three are known to regulate the concentration of biologically encoded amino acids glycine, lysine, and glutamine. While some comparative genomics studies of riboswitches focusing on their gross distribution across different bacterial taxa have been carried out recently, systematic functional annotation and analysis of lysine riboswitches and the genes they regulate are still lacking. We analyzed 2785 complete bacterial genome sequences to systematically identify 468 lysine riboswitches (not counting hits from multiple strains of the same species) and obtain a detailed phylogenomic map of gene-specific lysine riboswitch distribution across diverse prokaryotic phyla. We find that lysine riboswitches are most abundant in Firmicutes and Gammaproteobacteria where they are found upstream to both biosynthesis and/or transporter genes. They are relatively rare in all other prokaryotic phyla where if present they are primarily found upstream to operons containing many lysine biosynthesis genes. The genome-wide study of the genetic organisation of the lysine riboswitches show considerable variation both within and across different Firmicute orders. Correlating the location of a riboswitch with its genomic context and its phylogenetic relationship with other evolutionarily related riboswitch carrying species, enables identification and annotation of many lysine biosynthesis, transporter and catabolic genes. It also reveals previously unknown patterns of lysine riboswitch distribution and gene/operon regulation and allows us to draw inferences about the possible point of origin of lysine riboswitches. Additionally, evidence of horizontal transfer of riboswitches was found between Firmicutes and Actinobacteria. Our analysis provides a useful resource that will lead to a better understanding of the evolution of these regulatory elements and prove to be beneficial for exploiting riboswitches for developing targeted therapies.

## Introduction

RNA regulators like riboswitches have been found to play a significant role in the regulation of gene expression in many prokaryotes as well as some fungi and plants [1–3]. They are cis-regulatory elements that are present in the 5’ untranslated regions (UTRs) of the mRNA, bind to specific ligands and control the expression of downstream genes that are involved in either the biosynthesis or transport of those ligands. The riboswitch architecture is defined by a highly conserved aptamer domain that provides a binding site for the ligand and an expression platform that changes conformation on ligand binding and thereby regulates gene expression by a variety of mechanisms that include transcription or translation termination or a combination of those two effects [4]. Nearly forty different classes of riboswitches have been discovered till date, which are distinguished by the ligand that binds to the aptamer domain. They have also been found to regulate genes and operons in a large number of pathogenic bacteria and are therefore ideal as drug targets [5,6]. Riboswitches can also be targeted by antisense oligonucleotides that can bind to sequence segments, with important structural characteristics, to suppress the expression of the genes/operons which are crucial for the bacterial essential metabolic pathways. Natural as well as synthetic compounds that are designed to mimic riboswitch-binding metabolites and possessing anti-microbial properties have been reported for thiamine pyrophosphate (TPP) [7], lysine [8], purine [9] and flavin mononucleotide (FMN) [10] riboswitches. Artificial riboswitches have also been engineered to control gene expression through binding of natural metabolites to them [11–14].

An understanding of the regulatory role of riboswitches along with our ability to manipulate them relies on their accurate identification and on the information about the pattern of riboswitch distribution, the nature of genes they regulate and the latter’s role in metabolism and transport. Several methods [15–20] have been developed for computational detection of riboswitches. Among them, covariance models (CMs) have been the most influential and widely used method of riboswitch detection because it considers both sequence and structural conservation in the aptamer domain [21]. Rfam database is dependent on CMs for annotation of ncRNAs [22]. But while searching genomic sequences with CMs, the E-value calibration step is needed which takes a large amount of computational time. By exploiting the high level of sequence conservation of the aptamer domain in each class of riboswitch, we had developed a pHMM-based model for fast and accurate detection of riboswitches [23]. Recently, a web-server called the Riboswitch Scanner [24] was developed to provide researchers with a user-friendly tool for detecting riboswitches and even T-box RNA’s with high sensitivity and specificity from both partial and complete genomic sequences. Comparative genomics analysis [25–28] can yield insights into riboswitches by revealing genomic patterns of riboswitch-based regulation and the potential new functional role of riboswitch-regulated genes in the transport of the corresponding ligand. Detailed phylogenetic analysis of riboswitch distribution can also shed light on the evolutionary history of riboswitches and suggest possible points of origin of these ancient regulatory elements which may have existed in a primordial RNA world [29].

Among the different classes of riboswitches, only three known riboswitch classes have been found to sense genetically encoded amino acids glycine and lysine. Lysine is an essential amino acid for all organisms but it’s only synthesized *de novo* in bacteria and plants. Lysine is mainly synthesized via the diaminopimelate (DAP) pathway in bacteria [30] although a few bacterial species exploit the α-aminoadipate pathway for lysine biosynthesis [31, 32]. The lysine biosynthesis pathway is essential for cell wall biosynthesis, sporulation, stress response, and bacterial virulence [33–35]. Moreover, the absence of lysine biosynthesis pathway in humans suggests that there are advantages of using riboswitches regulating lysine biosynthesis genes in pathogenic bacteria as anti-microbial drug targets. This can be done by developing artificial ligands (following a method developed by Howe et al [10]) that mimic lysine and therefore bind to the aptamer domain of lysine riboswitches. Increase in concentration of such artificially synthesized molecules can turn off expression of essential lysine genes in bacteria thereby killing the bacteria even though actual lysine concentration may be quite low.

Using a comparative genomics approaches *Rodionov et al* [30] first identified lysine riboswitches upstream to various lysine biosynthesis and transporter genes in some bacterial species. By analyzing the genomic location of riboswitches, they could identify lysine biosynthesis and three new transporter genes. However, their analysis was restricted to just 37 bacterial genomes where they identified 71 potential riboswitches that they called Lys elements. With the advent of high-throughput sequencing technologies, vast amounts of complete genome sequence data are now available for many more bacterial species. Although Rfam database [22] provides the CM hits in complete and draft bacterial genome sequences that reveal the gross distribution of riboswitches, Rfam does not provide any gene-specific riboswitch distribution. Hence, the regulatory patterns of any class of riboswitches cannot be inferred from Rfam. In this study, our aim is to carry out a comparative genomics and phylogenomic analysis of lysine riboswitch distribution across all prokaryotic phyla with the aim of understanding the pattern of lysine regulation via riboswitches. By mapping out the gene-specific phylogenetic distribution of lysine riboswitches, we could identify and annotate the diverse set of genes or operons that are regulated by these riboswitches. In some instances, we even found evidence of horizontal transfer of lysine riboswitches between species belonging to different bacterial phylum, along with the genes they regulate. Our work provides a comprehensive analysis of the distribution and regulatory patterns of lysine riboswitches in the bacterial world.

## Results and discussion

### Patterns of regulation of lysine biosynthesis genes by lysine riboswitch

**Fig 1:**
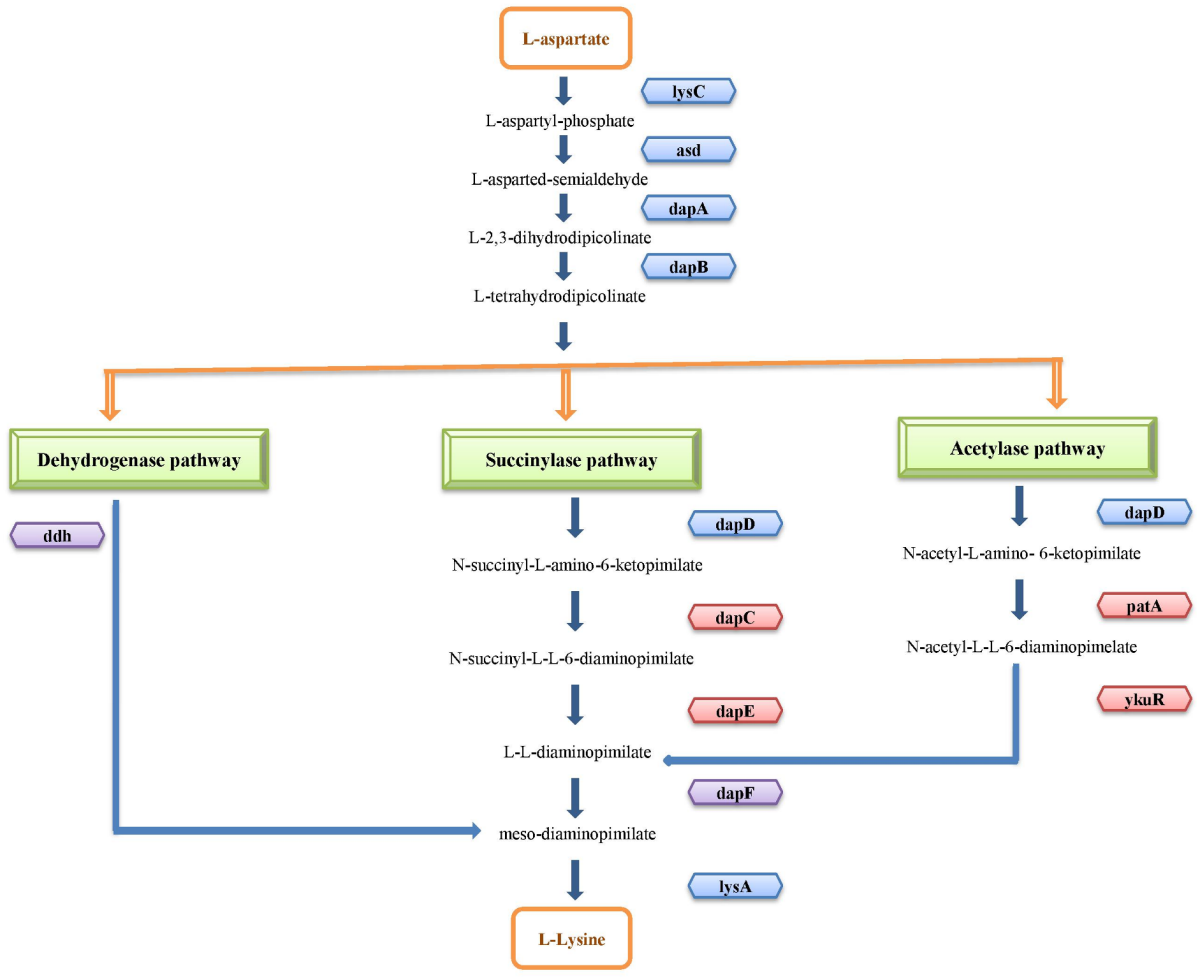
Lysine biosynthesis DAP pathway. Lysine biosynthesis genes regulated by lysine riboswitches are shown in blue. Lysine biosynthesis genes which are not regulated by lysine riboswitches in any organisms are shown in red. Lysine biosynthesis genes found to be regulated by riboswitches in very few instances are highlighted in purple.

Lysine is an essential amino acid which is synthesized *de novo* in the bacteria via DAP pathway [30] which diverges into three routes, as shown in Fig 1, depending on the bacterial species, namely the succinylase, acetylase (commonly found in several Bacillus species) and dehydrogenase pathways. In some organisms, the dapD gene belonging to the succinylase and acetylase pathways is present within an operon that consists of other lysine biosynthesis genes regulated by riboswitches. Exceptions are found in *Brevibacillus brevis* and *Colwellia psychrerythraea* where the lysine riboswitch is found upstream to an independently regulated dapD gene. In the case of dehydrogenase route, only *Tepidanaerobacter acetatoxydans* has a riboswitch in the 5’UTR of the ddh gene that is unique to that route. All the species of Thermotogae and two species of Fusobacteia, namely *Sebaldella termitidis* and *Leptotrichia buccalis*, have lysine riboswitches upstream to a conserved operon comprising of lysine biosynthesis genes that also contains the dapF gene. No riboswitches were found upstream to a standalone dapF gene in any species. In the subsections below, we discuss the phylogenetic distribution of lysine riboswitches found upstream to different lysine biosynthesis genes or operons in different classes of bacteria.

### Firmicutes

**Fig 2:**
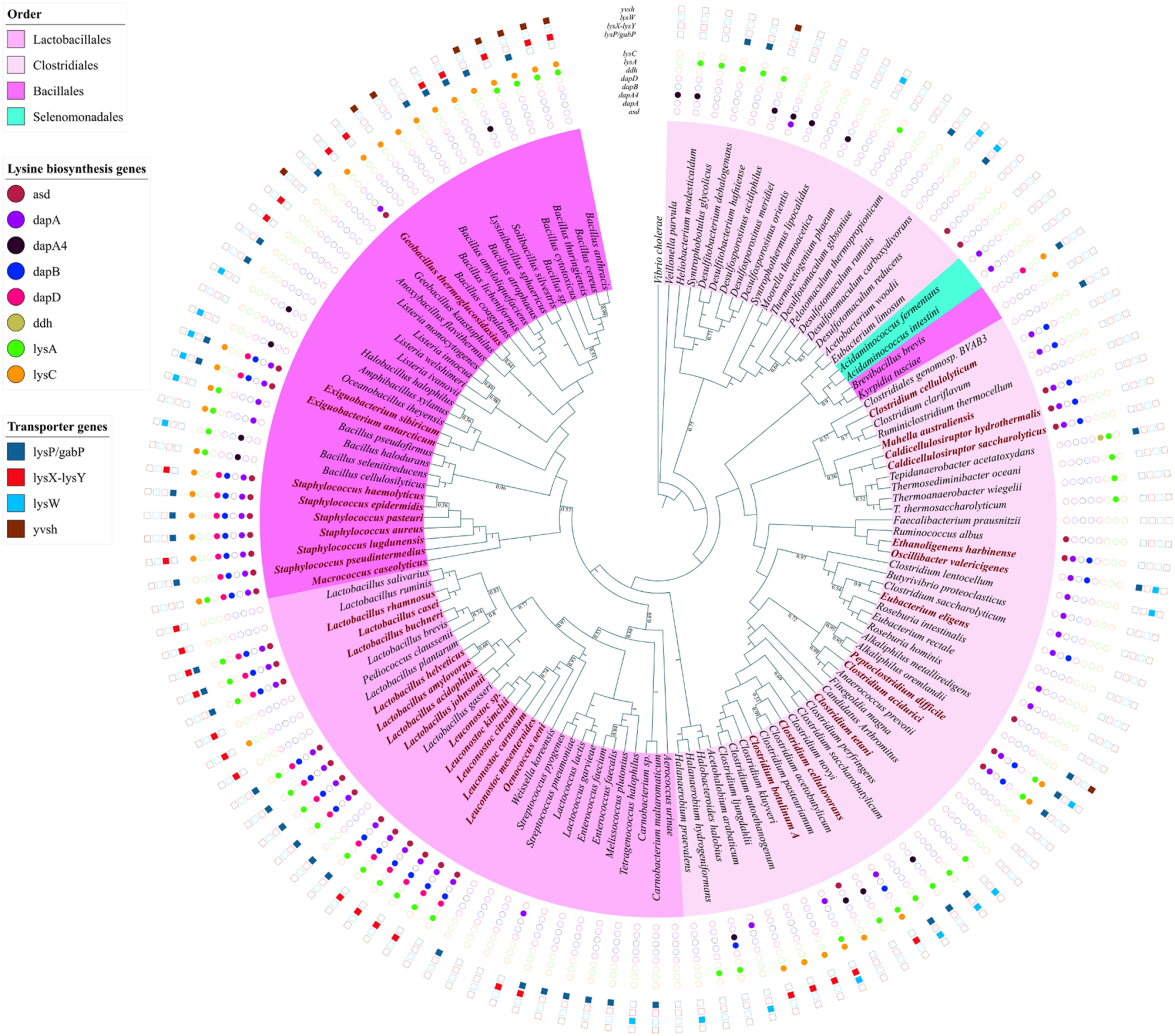
Phylogenomic distribution of riboswitch regulated lysine biosynthesis genes/operons and lysine transporter genes in the phylum Firmicutes. The different biosynthesis and transporter genes are color-coded according to a scheme specified in the figure legend. Filled circles indicate both the riboswitch and the gene are present, unfilled circles indicate that the riboswitch is absent but the corresponding gene is present. Species highlighted in bold, maroon color possess riboswitches upstream to operons containing multiple biosynthesis genes all of which are represented by color-coded filled circles. Filled squares indicate both the riboswitch and the gene are present, unfilled squares indicate that the riboswitch is absent but the corresponding transporter gene is present.

Lysine riboswitches are predominantly found in Firmicutes with species belonging to the orders Bacillales, Lactobacillales and Clostridiales containing the largest number of such riboswitches. S1 File shows the gross distribution of lysine riboswitches found upstream to biosynthesis and transporter genes while Fig 2 show the lysine biosynthesis and transporter gene-specific distributions respectively. It is evident from these figures that the lysine riboswitch distribution (discussed below) can vary significantly even across distinct species within the same bacterial orders.

### Bacillales

In the gram-positive bacteria from the Bacillales order, lysine biosynthesis genes were found either as single genes or within an operon containing several biosynthesis genes. To understand the underlying reasons behind this variation we analyzed the genomic context of lysine riboswitches and the operon structure of riboswitch-regulated genes in all the species belonging to a specific order. Table 1 shows the structure of operons regulated by riboswitches in different prokaryotic species. We hypothesize that a model of operon formation and disintegration proposed earlier [36–38] can influence lysine riboswitch-based gene regulation in different species of firmicutes. The model enables us to understand how the operon structure may have evolved as a result of insertion/deletion of genes from an operon and splitting of the operon. The general structure of the model is given in S2 File while specific cases pertaining to different prokaryotic phyla are discussed below.

**Table 1:** Lysine riboswitch-regulated operons.

Phylum	Order	Species	Riboswitch regulated operons
Firmicutes	Bacillales	*Macrococcus caseolyticus*	lysC-asd-dapA-dapB-dapD-amino hydrolase-alr-lysA
		*Staphylococcus lugdunensis*	lysC-asd-dapA-dapB-dapD
		*Staphylococcus pasteuri*	
		*Staphylococcus epidermidis*	
		*Staphylococcus haemolyticus*	
		*Staphylococcus pseudintermedius*	asd-dapA-dapB-dapD
		*Exiguobacterium antarcticum*	lysC-asd-dapA-dapB-dapH-aminotransferase
		*Exiguobacterium sibiricum*	
		*Geobacillus thermoglucosidasius*	lysC-asd-dapA
	Lactobacillales	*Lactobacillus rhamnosus*	lysA-dapD-dapA-dapB-asd
		*Lactobacillus casei*	
		*Lactobacillus buchneri*	
		*Lactobacillus helveticus*	
		*Lactobacillus amylovorus*	
		*Lactobacillus acidophilus*	
		*Lactobacillus johnsonii*	
		*Oenococcus oeni*	
		*Leuconostoc sp*.	lysA-dapD-dapA-dapB *and* alr-asd
		*Leuconostoc kimchi*	
		*Leuconostoc citreum*	
		*Leuconostoc carnosum*	
		*Leuconostoc mesenteroides*	
	Clostridiales	*Clostridium acidurici*	lysA-lyC-asd-dapA-dapB
		*Peptoclostridium difficile*	lysA-asd-dapA-dapB
		*Clostridium tetani*	lysA-cyclic beta 1–2 glucan synthetase
		*Clostridium cellulovorans*	lysC-lysA and dapA-dapB
		*Clostridium botulinum A*	lysC-dapA-lysA
		*Clostridium cellulolyticum*	dapA-dapB
		*Eubacterium eligens*	
		*Ethanoligenens harbinense*	asd-dapA-dapB- acetyltransferase
		*Caldicellulosiruptor saccharolyticus*	
		*Caldicellulosiruptor hydrothermalis*	
		*Mahella australiensis*	asd-dapA-dapB
		*Oscillibacter valericigenes*	dapA-dapB-acetyltransferase
Gammaproteobacteria	Alteromonadales	*Pseudoalteromonas haloplanktis*	dapA-dapD-lysA-hypothetical1-hypothetical2
		*Pseudoalteromonas sp*.	
Deltaproteobacteria	Bdellovibrionales	*Bacteriovorax marinus*	lysC-dapB-dapA-dapD-lysA
Thermotogae	Thermotogales	*Mesotoga prima*	asd-dapF-dapA-dapB-dapD-lysC-aminotransferase-lysA
		*Kosmotoga olearia*	asd-dapF-dapA-dapB-dapD-lysC-aminotransferase
		*Thermosipho melanesiensis*	
		*Thermosipho africanus*	
		*Marinitoga piezophila*	asd-lysA-dapA-dapB-dapD-lysC- aminotransferase-amidohydrolase
		*Petrotoga mobilis*	dapA-dapB-dapD-lysC- aminotransferase-amidohydrolase
		*Thermotoga neapolitana*	asd-dapF-dapA-dapB-dapD-lysC-lysA-amidohydrolase
		*Thermotoga maritima*	
		*Thermotoga sp*.	
		*Thermotoga petrophila*	
		*Thermotoga naphthophila*	
Fusobacteria	Fusobacterales	*Sebaldella termitidis*	lysA-lysC-dapF-hypothetical-dapA-asd
		*Leptotrichia buccalis*	lysA-lysC-dapF
Actinobacteria	Coriobacteriales	*Atopobium parvulum*	lysA-dapA-dapB
		*Slackia heliotrinireducens*	lysA-dapA-dapB-dapD
Acidobacteria	Solibacterales	*Candidatus Solibacterusitatus*	asd-lysC

**Fig 3:**
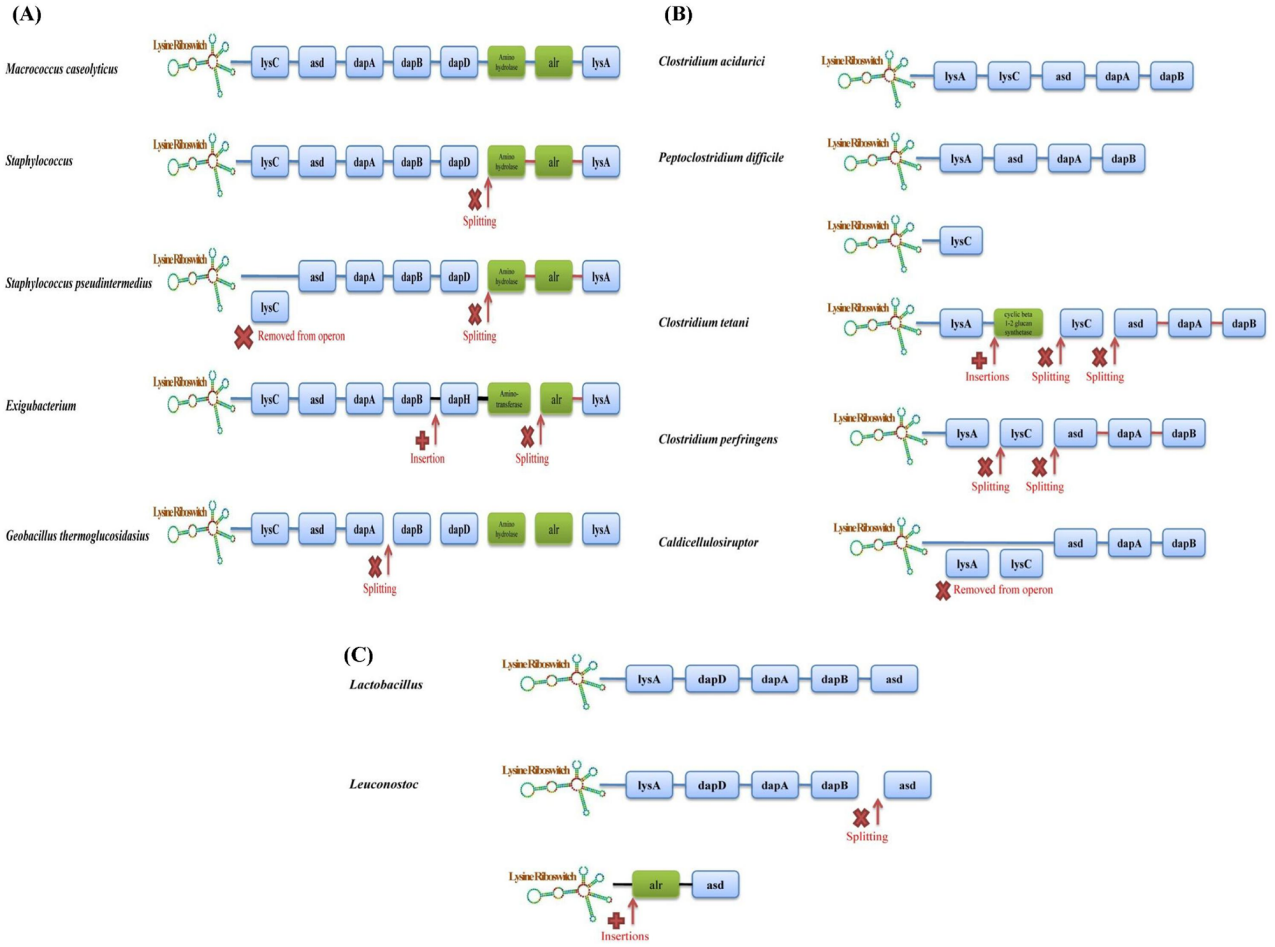
Genome rearrangement and operon organizations in (A) Bacillales (B) Clostridiales and (C) Lactobacillales. Blue and green filled rectangles represent lysine biosynthesis genes and those genes that are not involved in lysine biosynthesis, respectively. Red cross marks locations where an operon has split or a gene has been removed from an operon. A red plus sign marks location where one or more genes have been inserted into the operon. For cases where only the genus name is provided, all the species of that genus have the same operon structure.

**Fig 4:**
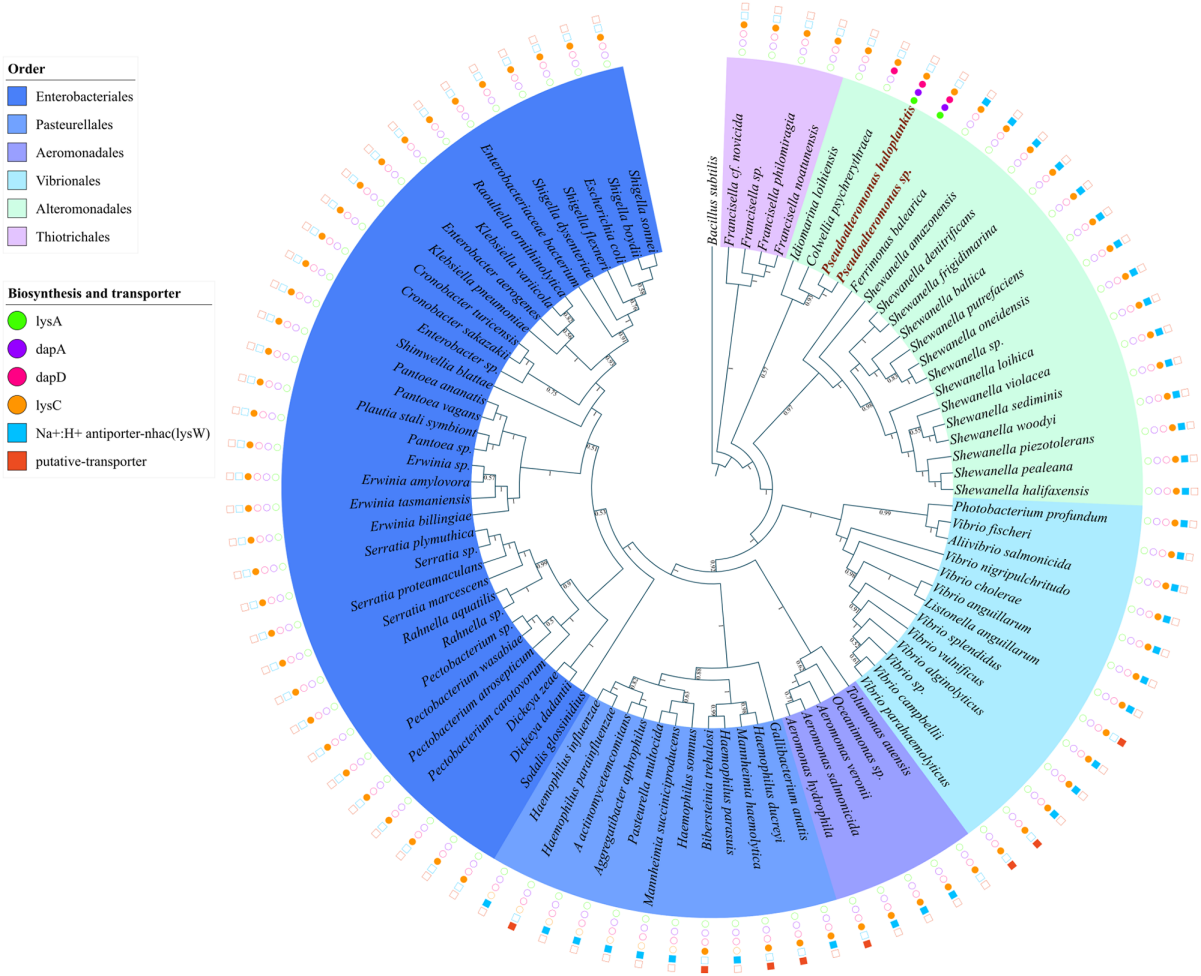
Phylogenomic distribution of riboswitch regulated genes and operons involved in lysine biosynthesis and transport in the phylum Gammaproteobacteria. Filled shapes (circle for biosynthesis and square for transporter genes) indicate both the riboswitch and the gene are present, unfilled shapes indicate that the riboswitch is absent but the corresponding gene is present. Species highlighted in bold maroon color possess a riboswitch upstream to an operon containing multiple biosynthesis genes all of which are represented by color-coded filled circles. The color-coding scheme is specified in the figure legend.

Fig 3(A) represents the organization of operons in varied species of Bacillales order. *Macrococcus caseolyticus* has the largest operon (lysC-asd-dapA-dapB-dapD-amino hydrolase-alr-lysA) which contains six lysine biosynthesis genes along with two other genes. It is also noteworthy that this species is one of the earliest branching species in this order. *Macrococcus caseolyticus* cluster with the species of the genus staphylococcus in the phylogenetic tree forming a clade seen in Fig 2. In phylogenetically related sister species belonging to the *Staphylococcus* genus, the original operon most likely disintegrated after the insertion of a functionally unrelated amino hydrolase gene just before the alr gene (in the common ancestor of the *Staphylococcus* genus) leading to the formation of two new operons (as depicted in Fig 3A) of which only the former is regulated by the lysine riboswitch. An exception to this pattern is only found in *S*. *pseudointermedius* where the first operon has further disintegrated into the riboswitch-regulated operon asd-dapA-dapB-dapD and a single lysC gene that is not regulated by a riboswitch. In the species of the *Exigubacterium* genus, two new genes are inserted into the operon after dapB gene, the lysine biosynthesis gene dapH and an amino transferase which is not a biosynthesis gene most probably leading to the splitting of the operon as shown in Fig 4A. The splitting can be understood to be a consequence of the insertion of the functionally unrelated amino transferase gene since unrelated genes in the operon can exhibit different gene expression patterns [38]. Similarly, in *Geobacillus thermoglucosidasius*, the operon consists of only the first three genes lysC-asd-dapA which are regulated by lysine riboswitch while the rest of the operon disintegrated and are present as single genes not regulated by any riboswitch. It is also instructive to note that the ddh biosynthesis gene is not a member of any operon and neither is a lysine riboswitch found upstream to this gene in Bacillales. In almost all species belonging to this order that has been represented in the tree, a lysine riboswitch if not present in the upstream of a biosynthesis gene can be found in the upstream of one or more transporter genes as discussed in a subsequent section. All these examples suggest that even though significant genomic reorganization can occur across related species, the riboswitch found upstream to the gene/operon likely predated the genomic reorganization events.

### Clostridiales

The Clostridiales are a highly polyphyletic class of Firmicutes and the distributions of lysine riboswitch in Clostridiales are quite fragmented as well. Lysine riboswitches are present in the upstream of an individual species’ lysine biosynthesis genes or an operon comprising of several biosynthesis genes. In Clostridiales, riboswitches found upstream to operons of biosynthesis genes are scattered throughout the order but with variable operon structure. *Clostridium acidurici* has the largest operon of lysine biosynthesis genes consisting of lysA-lyC-asd-dapA-dapB, which is regulated by a riboswitch. In the closely related species *Peptoclostridium difficile*, the lysC gene has separated from the operon (see Fig 3B) but is present as a single riboswitch-regulated gene along with the shorter riboswitch-regulated operon lysA-asd-dapA-dapB. In *Clostridium tetani*, the operon has split probably following the insertion of a functionally unrelated cyclic beta 1–2 glucan synthetase gene after lysA (Fig 3B) resulting in the riboswitch-regulated operon lysA-cyclic beta 1–2 glucan synthetase, followed by a single lysC gene and the operon asd-dapA-dapB, neither of which is regulated by a riboswitch. Cyclic beta 1–2 glucan synthetase is a potential virulence factor in many pathogenic bacteria such as Brucella, Agrobacterium and it is important for intracellular survival in the host [39, 40] suggesting the riboswitch upstream to this operon as a viable drug target. In *Clostridium cellulovorans*, the asd gene has split from the largest operon leading to the formation of two new operons lysC-lysA and dapA-dapB both of which are regulated by lysine riboswitches unlike the single asd gene. In *Clostridium botulinum*, extensive genome rearrangement seems to have occurred within the operon together with the deletion of the dapB and asd genes from the operon leading to the lysC-dapA-lysA operon which is regulated by lysine riboswitch. In *Clostridium cellulolyticum* the lysA, lysC and asd gene are absent from the riboswitch-regulated operon which consists of dapA-dapB genes only. In *Caldicellulosiruptor* and *M*. *australiensis*, the lysA and lysC gene are absent from the operon but present as single genes neither of which is regulated by a riboswitch. The riboswitch carrying operon, therefore, consists of asd-dapA-dapB genes. In other riboswitch containing Clostridiales species, a riboswitch is present upstream to one or more of the biosynthesis genes present in the largest operon.

### Lactobacillales

Some Lactobacillus species like *Lactobacillus plantarum*, *Lactobacillus brevis*, *Lactobacillus ruminis*, *Lactobacillus salivarius* and Enterococcus species like *Enterococcus faecalis* and *Enterococcus faecium* and species from Carnobacterium, Tetragenococcus, Melissococcus do not possess lysine riboswitches upstream to biosynthesis genes but carry a lysine riboswitch upstream to a transporter gene. The lysine biosynthesis pathway is missing in *Lactobacillus gasseri* and *Weissella koreensis* but these organisms also regulate lysine concentration using lysine riboswitches upstream to transporter genes. *Streptococcus pyogenes* does not possess the lysine biosynthesis pathway and neither does it possess any lysine riboswitches.

Some species belonging to the genus *Lactobacillus* and *Oenococcus*, have a conserved operon comprising of lysine biosynthesis genes lysA-dapD-dapA-dapB-asd that is regulated by a lysine riboswitch. In *Leuconostoc*, similar patterns are found (see Table 1) but the insertion of the functionally unrelated alr (alanine racemase) gene in the operon after the dapB gene causes the split of the operon as shown in Fig 3C. Nevertheless, the splitting of the operon cannot account for the presence of a riboswitch upstream to the alr-asd operon which must have appeared after the operon splitting event in the common ancestor of *Leuconostoc*s. In all the genomes where the operon splits after the inserted gene unrelated to Lysine biosynthesis, the corresponding gene is a present as a single copy. It also has a distinct functional role unrelated to lysine biosynthesis. Even though the mode of regulation of that gene after splitting from the original operon is not clear but it is likely that the splitting is correlated with the need to regulate that gene differently from lysine biosynthesis genes. In *P*. *claussenii*, even though the operon (which includes two additional genes not associated with lysine biosynthesis) is present, it is not regulated by a riboswitch which is found upstream to a single lysC gene. In contrast in the closely-related sister species *L*. *plantarum* and *L*. *brevis*, no riboswitch can be found upstream to any of the biosynthesis genes. This pattern provides further evidence that riboswitch regulation of biosynthesis genes evolved in diverse ways along different branches.

### Gammaproteobacteria

In Gammaproteobacteria (Fig 4), lysine riboswitches are mainly found upstream to a single lysC gene. Exceptions are Pseudoalteromonas where a lysine riboswitch was found in the upstream of a single lysC gene but another lysine riboswitch is also present upstream to an operon consisting five genes where three of them are lysine biosynthesis genes and the remaining two are hypothetical proteins (see Table 1). The latter by their presence only in Pseudoalteromonas must have originated in the common ancestor of that genus. In *E*. *coli*, lysine riboswitches modulate access to the ribosome-binding site of the lysC transcript and interact with the RNase E protein regulating the translation initiation of the lysC transcript [41–43]. In the presence of lysine, the lysine riboswitch changes its conformation in such way as to enable RNase E to access the cleavage sites and degrade the lysC transcript. In the absence of lysine, the RNase E cleavage sites are sequestered by the riboswitch, preventing transcript degradation by RNase E which promotes the production of functional lysC. From Fig 4 we can see that this regulatory system is well conserved within species of *Enterobacteriales* and widespread in several other orders of Gammaproteobacteria. Therefore, in Gammaproteobacteria, the mode of regulation of DAP pathway genes by lysine riboswitches is different from that in Firmicutes where the lysine riboswitches mainly regulate the transcription initiation of lysine biosynthesis genes.

### Other bacteria

**Fig 5:**
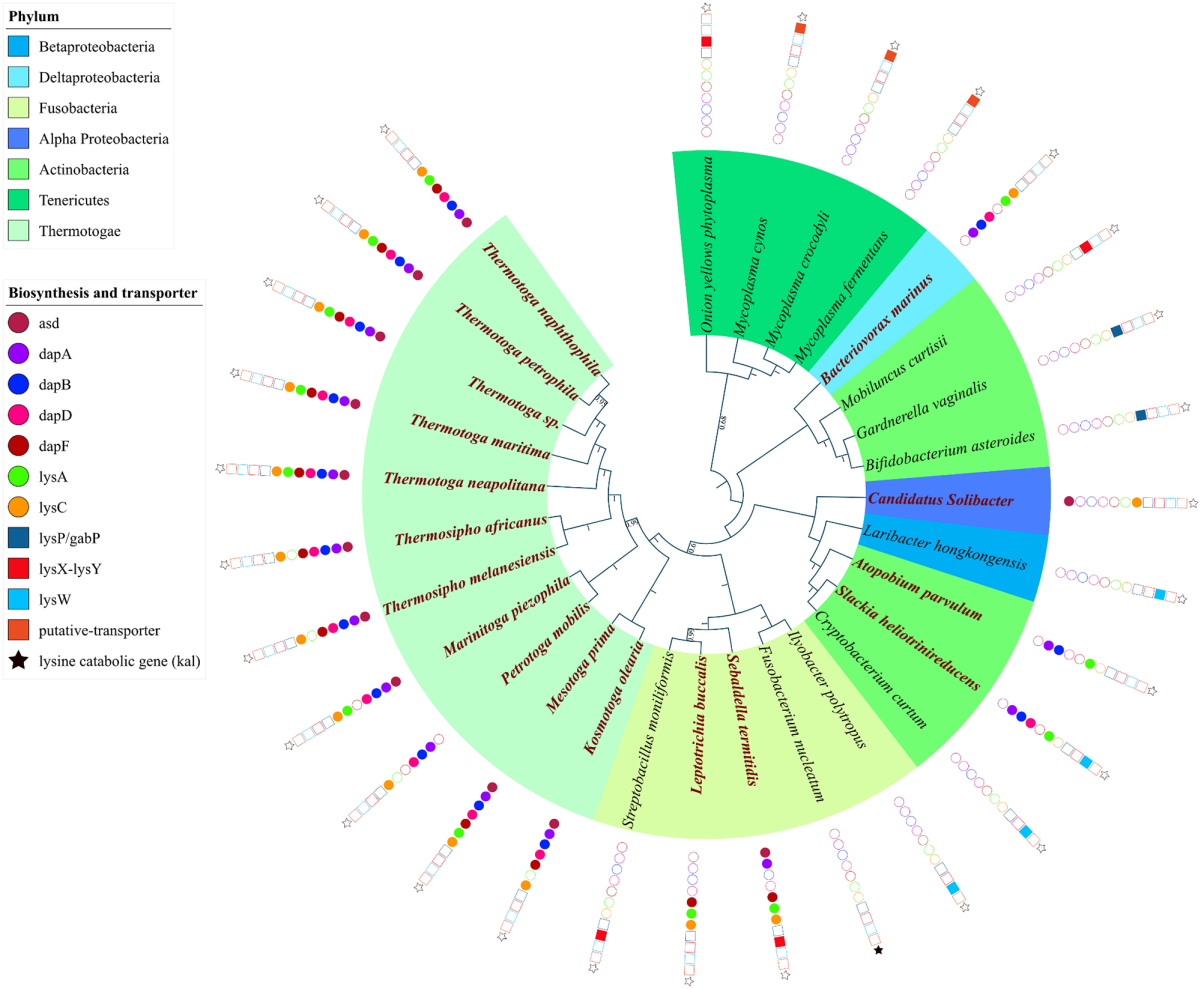
Phylogenomic distribution of riboswitch regulated genes and operons involved in lysine biosynthesis and transport in all other phylum excluding Firmicutes and Gammaproteobacteria. Filled shapes (circle for biosynthesis, square for transporters genes and star for lysine catabolic genes) indicate both the riboswitch and the gene are present; unfilled shapes indicate that the riboswitch is absent but the corresponding gene is present. Species highlighted in bold maroon color possess a riboswitch upstream to an operon containing multiple biosynthesis genes all of which are represented by filled circles.

In this category shown in Fig 5, riboswitches upstream to lysine biosynthesis genes are found mainly in few species belonging to the phylum Tenericutes, Actinobacteria, Fusobacteria, Thermotoga, Acidobacteria, Betaproteobacteria, and Deltaproteobacteria. In all these cases, the lysine biosynthesis genes are typically organized in an operon but the constituent genes in the operon can vary as shown in Table 1. For example, in several species of Thermotogae phylum, the lysine riboswitch is found upstream to a conserved operon which consists of most of the lysine biosynthesis genes (see Table 1 for the operon structure). However, the insertion and/or removal of certain genes from the operon and reorganization of the operon structure are observed across this as well as other phyla belonging to this category.

### Transporter genes regulated by lysine riboswitch

Lysine riboswitches are also found upstream to various lysine transporter genes that are scattered across many prokaryotic phyla but are most widespread in Firmicutes. Our systematic analysis enabled us to identify many new instances of lysine riboswitches upstream to known lysine transporter genes. Table 2 gives the COG ID of every riboswitch-regulated lysine transporter gene. The lysine-specific permease, the lysP gene is a known candidate gene for lysine transport in Firmicutes [30]. Based on the presence of lysine riboswitch, we could identify a lysine transporter gene, which is functionally annotated as gamma-aminobutyrate-permease (lysP/gabP) that has not been previously implicated in lysine transport. They are widespread primarily across Bacillales and Lactobacillales but also found in a few Clostridiales and the Actinobacteria species *Bifidobacterium asteroides*. Analysis of GO molecular function of the gabP transporter reveals its role in amino acid transmembrane transport. NCBI-CDD (conserved domains database) searches highlighted the presence of a highly-conserved lysine-specific transporter (lysP) domain in the sequences of gabP. Further, high sequence similarity and a similar number of transmembrane domains within the gabP and lysP gene indicates gabP can act as a substitute for the lysP genes in some organisms. After this work was initiated, NCBI has changed the annotation of several lysP genes to gabP. In Figs 2 and 5, we have therefore labeled this gene lysP/gabP. Many more instances of other previously known [30] lysine transporter genes such as yvsH and lysW were found to be scattered across Firmicutes (see Fig 2). Among these, the lysW riboswitch is the most widely distributed across diverse prokaryotic phylum such as Firmicutes, Gamma, and Betaproteobacteria. The protein encoded by lysW gene contain 11 candidate transmembrane domains and belongs to a unique protein family, which is a part of the nhaC Na+: H+ antiporter superfamily and is implicated in H^+^ transport. Hence it was suggested that lysine riboswitches found upstream of these genes are also involved in maintaining cellular pH [44].

**Table 2:** COG ID’s of lysine transporter genes. The first two genes have the same COG ID but are sometimes annotated as lysP and at other times as gabP which also contains a lysP domain.

COG ID	Gene name	Gene product description
COG0833	lysP	Lysine-specific permease
COG0833	gabP	gamma-aminobutyrate-permease
COG1757	lysW	na+ h+ antiporter (nhaC)
COG0531	Yvsh	Arginine:ornithine antiporter
COG0834	lysX-lysY	ABC-type amino acid transporter

The lysX-lysY gene was tentatively identified [30] as a potential lysine transporter based on the presence of the lys element upstream to this gene in just 3 species (*L*. *gasseri*, *O*. *oeni* and *E*. *faecalis*) belonging to the Lactobacillales order. However, our analysis does not corroborate those claims since we found a Lysine riboswitch upstream to the lysP/gabP gene instead of the lysX-lysY system in those 3 species. Nevertheless, we detected many lysine riboswitches upstream to the lysX-lysY gene to validate its candidature as a bonafide Lysine transporter. As shown in Figs 2 and 5, the lysX-lysY riboswitch scattered across Firmicutes, Actinobacteria, Fusobacteria and are involved in regulating lysine transport wherever lysW, lysP, and yvsH are absent. In fact, the lysX-lysY gene shows significant similarity with the members of ATP-dependent ABC transporter superfamily [30]. A class of ABC transporter gene having high similarity with the lysX-lysY genes is annotated as glutamine ABC transporter in a few species of Lactobacillus (*L*. *acidophilus*, *L*. *amylovorus* and *L*. *garvieae*) and is also regulated by a lysine riboswitch. Therefore, certain species of bacteria utilize generic ATP dependent amino-acid transporters for lysine transport as well.

Lysine riboswitches were detected upstream to single hypothetical genes in a few species. The annotations of those hypothetical genes are not yet available. However, an examination of the phylogenetic tree indicates that such organisms share a close evolutionary relationship with other species containing a lysine riboswitch upstream to a transporter gene. This pattern suggests the possibility that those hypothetical genes might have a role in lysine transport. For example, in the order Pasteurellales, lysine riboswitch is found upstream to hypothetical genes in *Haemophilus parainfluenzae*, *Bibersteinia trehalosi*, *Haemophilus ducreyi* and *Mannheimia haemolytica*. But, other species of this order carry lysine riboswitch in the upstream of the lysW transporter gene. We found that those hypothetical proteins show significant sequence similarity with diverse types of transporter genes listed in the Transporter Classification DataBase (TCDB) in BLASTP searches. Moreover, these genes carry a conserved amino acid transporter domain in their sequences. These characteristics together with the detection of a lysine riboswitch upstream to these genes suggest that those hypothetical proteins might be considered as potential lysine transporters. Similarly, in the order Vibrionales, we have annotated the hypothetical proteins as transporters in *Vibrio parahaemolyticus*, *Vibrio campbellii* and *Vibrio splendidus*. Since we were unable to confirm the precise class of transporters, these genes were mapped onto the phylogenetic tree of gammaproteobacteria and other bacteria as putative transporters and denoted by deep orange squares in Figs 4 and 5.

In Tenericutes, *Onion yellow phytoplasma* has a lysine riboswitch upstream to a single ABC transporter gene (lysX-lysY system) as shown in Fig 5 but in a few closely related Mycoplasma species, a lysine riboswitch was found in the upstream of a gene whose product is oligoendopeptidase F (COG1164). Gene Ontology functional classification indicates the role of oligoendopeptidase F in amino acid transport and metabolism. Later using a computational approach pioneered by *Dam*, *P*. *et al*., [45] we were able to annotate an operon where the gene encoding oligoendopeptidase F clusters with a gene whose product is a putative intrinsic membrane protein that is conserved within the species of Mycoplasma. We subsequently found that both genes in this operon have conserved amino acid transporter domains and transmembrane domains in their sequences that point to their role as potential lysine transporters. We were not able to identify the specific class of transporter; hence this operon is represented in the phylogenetic tree of ‘other’ bacterial classes as putative transporters and denoted by deep orange squares in Fig 5.

### Regulations of lysine catabolic genes by lysine riboswitch

In certain species, lysine riboswitches are present in the upstream of lysine catabolic genes. Unlike lysine biosynthesis genes that are repressed by high lysine concentration, these catabolic genes are positively regulated [44] by the lysine riboswitch. These genes are involved in the L-lysine degradation via the acetate pathway and are therefore activated when lysine concentration is high. In *Clostridium sticklandii* one lysine riboswitch is located upstream to an operon consisting of two lysine catabolic genes ‘kal’ and ‘kce’. In *Fusobacterium nucleatum*, a lysine riboswitch is detected upstream of a single ‘kal’ gene depicted by a filled black star in Fig 5. In *Thermoanaerobacter tengcongensis* and *Thermoanaerobacter wiegelii*, a lysine riboswitch is present upstream to an operon beginning with the pspF3 transcriptional regulator gene and followed by kal and kce genes, which are involved in the lysine catabolism in Clostridium species. The structure-based energy minimization method of detection provided further confirmation of the presence of lysine riboswitches in *T*. *tengcongensis* and *T*. *wiegelii*.

### Horizontal riboswitch transfer (HRT)

**Fig 6:**
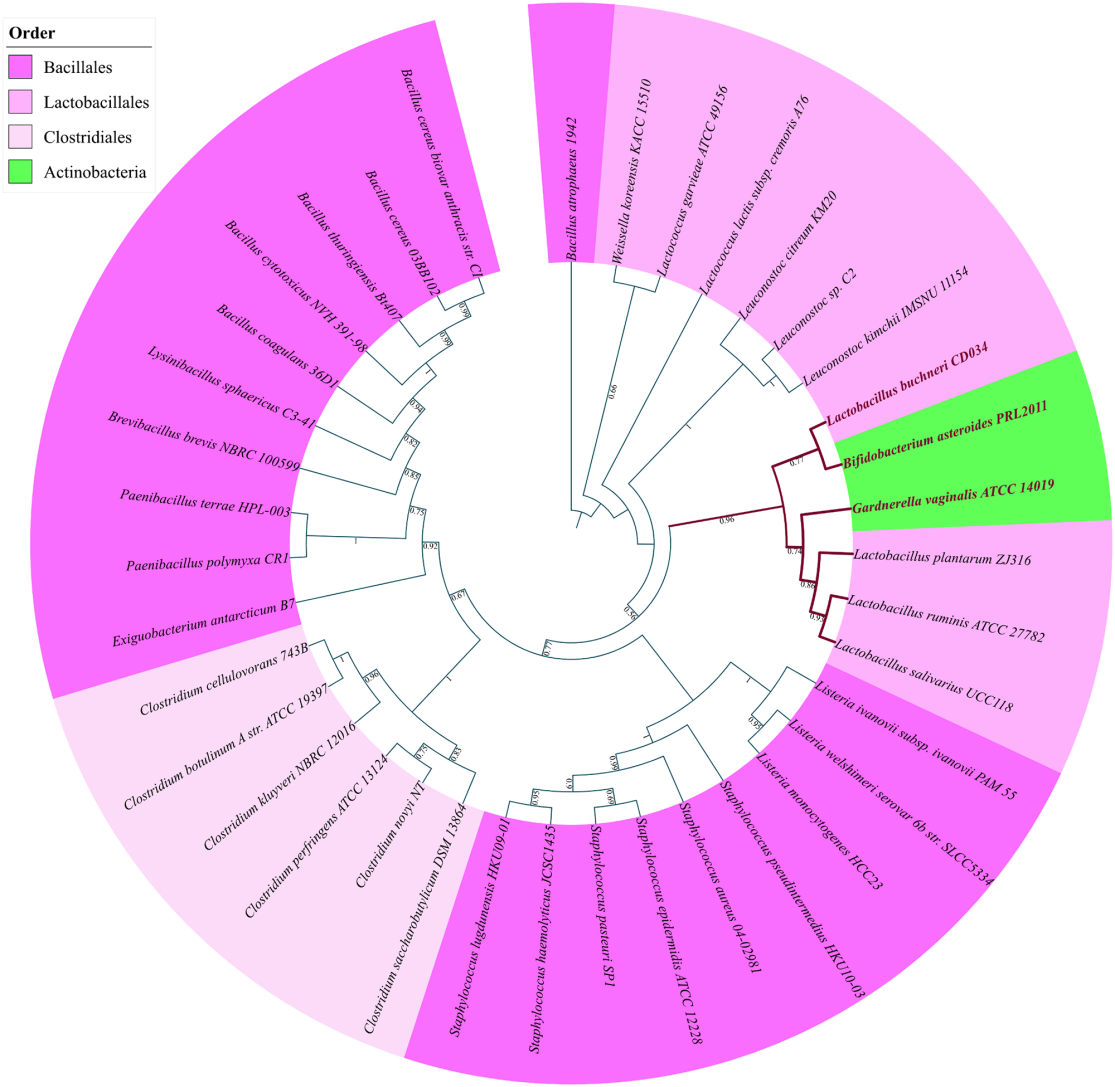
Phylogenetic tree of lysP/gabP transporter genes found in Firmicutes and Actinobacteria. Potential HRT event has occurred between species highlighted in maroon color.

Horizontal transfers of riboswitches along with the genes they regulate have been previously detected for the case of purine riboswitches [46]. We also found some evidence that suggests horizontal transfer of lysine riboswitches along with the regulated transporter genes among different prokaryotic orders. A lysine riboswitch upstream to the lysP/gabP transporter gene is found in many species of firmicutes (see Fig 2) but the gene along with the riboswitch is present in only two Actinobacerial species, *Gardnerella vaginalis ATCC 14019* and *Bifidobacterium asteroids PRL2011*. When a gene tree for lysP/gabP is constructed (see Fig 6), we find that *B*. *asteroids PRL2011*and *G*. *vaginalis ATCC 14019* not only clusters with organisms from the *Lactobacillales* order but also share the same clade (shown by maroon branches in Fig 6) with *L*.*buchneri CD034* (and a few other *Lactobacillales* species) all of which possess the lysP/gabP gene, thereby strongly suggesting to HRT of the lysP riboswitch along with the gene from the latter to the former.

**Fig 7:**
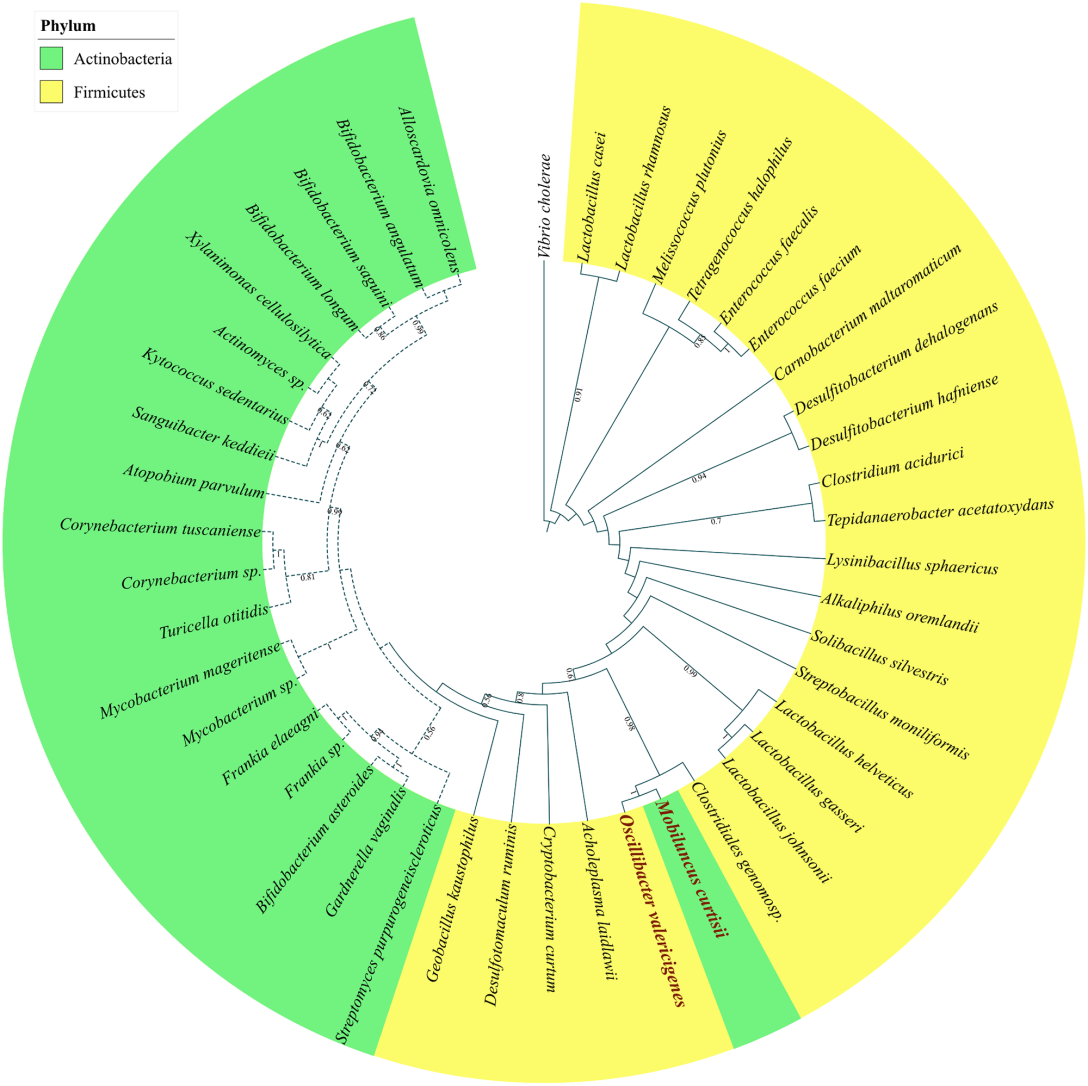
Phylogenetic tree of lysX-lysY genes found in Firmicutes and Actinobacteria. Dotted lines represent the clade where a riboswitch is not present upstream to the lysX-lysY gene. Potential HRT event has occurred between species highlighted in maroon color.

A gene tree of the LysX-lysY gene (Fig 7) shows evidence for HGT of the gene from *Oscillibacter valericigenes Sjm18-20* (Clostridiales) to *Mobiluncus curtisii ATCC 43063* (Actinobacteria). All the Actinobacterial species cluster together to form a clade with the exception of *M*. *curtisii* whose LysX-lysY gene clusters with *O*. *valericigenes* belonging to the Clostridiales order. It is also telling that *M*. *curtisii* is the only actinobacterial species that possess both the LysX-lysY gene as well as a riboswitch upstream to it.

Additional indications about horizontal transfer events associated with the lysP/gabP gene and the LysX-lysY gene from HGTree database [47] lends further support for HRT between *L*.*buchneri CD034* and *B*. *asteroids PRL2011* and between *O*. *valericigenes* and *M*. *curtisii*.

**Fig 8:**
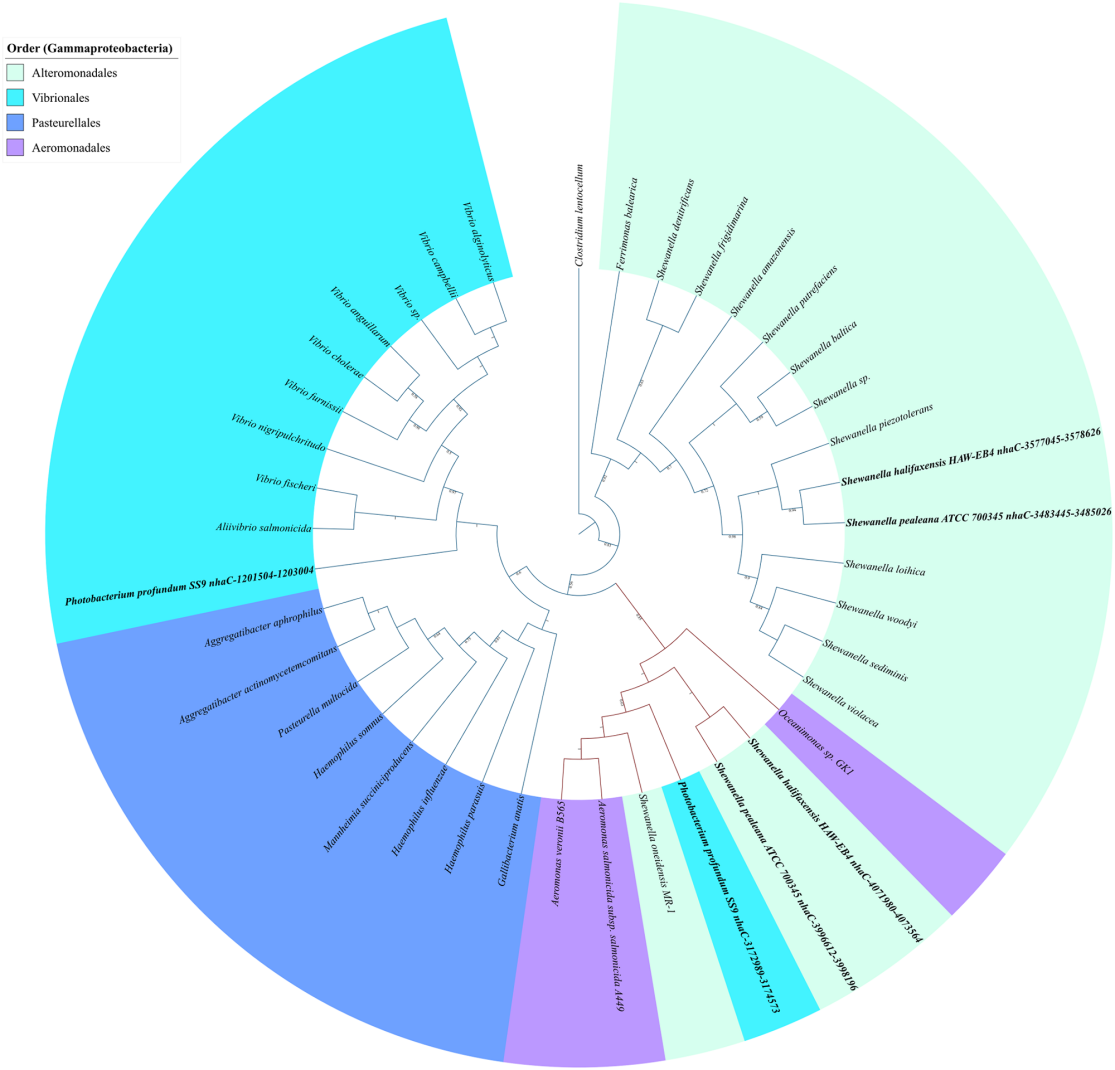
Phylogenetic tree of lysine riboswitch regulated lysW genes of gammaproteobacteria. The clade highlighted in the tree by maroon branches depicts the HRT event.

*Photobacterium profundum SS9*, *Shewanella halifaxensis HAW-EB4* and *Shewanella pealeana ATCC 700345* have two lysW genes and lysine riboswitch is found in the upstream of both genes. From the phylogenetic tree of the lysW gene, it is found that one copy of the lysW gene from *Shewanella halifaxensis HAW-EB4* and *Shewanella pealeana ATCC 700345* cluster with all other species of *Shewanella* except *Shewanella oneidensis MR-1*. Similarly, one copy of the lysW gene found in *Photobacterium profundum SS9* cluster with other species belonging to the *Vibrionales* order. However, the second copy of the lysW genes of *Photobacterium profundum SS9*, *Shewanella halifaxensis HAW-EB4* and *Shewanella pealeana ATCC 700345* form a distinct clade with *Shewanella oneidensis MR-1*, *Oceanimonas sp*. *GK1*, *Aeromonas salmonicida* and *Aeromonas veronii*, where the last three species belong to the *Aeromonadales* order. All members of this cluster are found in marine environments. Therefore, we hypothesize that second copy of the lysW gene of *Shewanella halifaxensis HAW-EB4*, *Shewanella pealeana ATCC 700345*, *Photobacterium profundum SS9*, along with the riboswitch upstream to it, arose as a result of a gene duplication event followed by divergence of the second copy relative to the original as it was subject to substantially less selection pressures. The greater similarity of the second copy of the lysW gene in *Shewanella halifaxensis HAW-EB4* and *Shewanella pealeana ATCC 700345* to the only copy of the same gene in *Shewanella oneidensis MR-1* suggests a common origin of the gene in these organisms. Moreover, the presence of the gene along with the riboswitch in three species belonging to the order *Aeromonadales* that cluster with the second lysW gene in the above *Shewanella* species (see Fig 8) suggests possible horizontal transfer from *Shewanella* to *Aeromonadales*. To further test this hypothesis, we performed the smart-blast of individual lysW genes from the species making up the second cluster. Smart-blast provides the five closest matches to high-quality sequences. The results (see S3 File) from the smart-blast indicates that for each of the lysW gene query for individual species from the second cluster, the *Shewanella oneidensis MR-1* lysW gene is returned every time within the top five matches along with other close relatives of the query species. When the second lysW gene of *Aeromonas salmonicida* and *Aeromonas veronii* is used as a query sequence, the identity with the lysW gene of *Shewanella oneidensis MR-1* is as high as 90%. On the contrary, when the first lysW gene of *Shewanella halifaxensis HAW-EB4*, *Shewanella pealeana ATCC 700345*, *Photobacterium profundum SS9* is used as a query, the second lysW gene does not appear within the top 5 matches. Since Smart-blast detects sequences that are highly similar to the query sequence, these results provide further evidence in support of our hypothesis.

## Conclusions

Our comparative genomics analysis and phylogenetic profiling of the distribution of lysine riboswitches in bacteria reveals some hitherto unknown patterns in riboswitch mediated gene regulation. By correlating the location of the riboswitches with their genomic context, we could associate many *generic* transporter genes with lysine transport. Lysine riboswitches were found to be widespread in Firmicutes with often multiple copies of lysine riboswitches found in many species belonging to this phylum. These riboswitches control a variety biosynthesis, transporter as well as some lysine catabolic genes. Almost all riboswitch-regulated biosynthesis genes (barring a small number of exceptions) fall in the common part of the DAP pathway. Regulation of such genes that appear early in the biosynthesis pathway achieves the greatest reduction in metabolic cost when lysine concentration in the cell is high. Since the Firmicute phylum is the earliest branching prokaryotic phylum [48, 49], the widespread presence of lysine riboswitches in Firmicutes suggest that its evolutionary origin can be traced back to the root of the Firmicute tree and lends support to the hypothesis that riboswitches are regulatory elements that could have emerged in a primordial RNA world [29, 50].

The gene composition of riboswitch-regulated operons can be understood on the basis of a model of operon formation and disintegration. The origin of some of the lysine riboswitches most likely predated the reorganization of the riboswitch-regulated genes within an operon due to reshuffling of gene order or disintegration of an operon. Nevertheless, such a model cannot explain the presence of multiple copies of riboswitches without invoking a viable mechanism of riboswitch duplication or emergence via evolution of the corresponding 5’UTR. As in the case of Purine riboswitches [46], we found evidence of riboswitch dispersal across distant prokaryotic phyla via HRT. Specifically, our analysis based on construction of gene trees indicate that the presence of a lysine riboswitch in some Actinobacterial species can be attributed to horizontal riboswitch transfer from Firmicutes along with the gene it regulates.

The phylogenetic profile of lysine riboswitches allows us to speculate on the possible evolutionary point of origin of these riboswitches. Even though the lysine riboswitch is widespread across Firmicutes, the gene-specific distribution is more fragmented thereby suggesting multiple points of origin for each gene-specific lysine riboswitch. However, the gene-specific distribution of lysine riboswitches is more uniform in Gammaproteobacteria with the riboswitch upstream to the lysC biosynthesis gene being found across all Gammaproteobacterial orders suggesting that the origin of this riboswitch can be traced back to the root of the Gammaproteobacterial tree. On the contrary, the lysW riboswitch appears to be of more recent origin since it is present only in species belonging to the orders *Alteromonadales*, *Aeromonadales*, *Vibrionales* and *Pasteurellales*. Nevertheless, the precise evolutionary point(s) of origin is difficult to infer in this case.

The phylogenetic profile also raises the issue of essentiality of lysine riboswitches for lysine regulation in prokaryotes. The ubiquitous presence of at least one lysine riboswitch in Firmicutes and Gammaproteobacteria is indicative of their critical role in lysine regulation. Nevertheless, we found 8 species belonging to this phylum (excluding *S*. *pyogenes* species discussed earlier) which does not contain a single lysine riboswitch (see S1 File). While the absence of any lysine riboswitch in these organisms cannot be explained based on their location in the phylogenetic tree, it suggests either the availability of alternative modes of lysine regulation in these organisms or the possibility that these organisms can function without regulating lysine concentration.

In view of the potential of lysine riboswitches as effective drug targets especially due to their presence in pathogenic bacteria and the absence of lysine biosynthesis pathway in humans, our comprehensive analysis of the distribution and genomic context of lysine riboswitches can be a helpful resource for more targeted genome-specific manipulation of lysine riboswitches.

## Methods

### Data acquisition

A total 2785 complete bacterial genome sequences were retrieved from the RefSeq database [51]. The genomes were classified into different phylum based on taxonomy. Operon information for all the genomes was retrieved from DOOR 2.0 (Database of prOkaryotic OpeRons) [52, 53] and ProOpDB (Prokaryotic Operon DataBase) [54]. After searching the lysine riboswitches in all those genomes, we found that all the strains from a species have the same pattern for the presence/absence of lysine riboswitches. Therefore, for removing the redundancy, we considered only one representative strain for a species. Shell scripts were used to extract and categorize the genomic data. BEDTools [55] were used to compare the sets of genomic features.

### Identification of lysine riboswitches

We used the lysine riboswitch specific pHMM from Riboswitch Scanner [24] and searched against the RefSeq complete bacterial genomes database. For further validations of lysine riboswitches detected by pHMM, we constructed the lysine riboswitch specific CM and searched against the pHMM predicted sequences. We detected 468 lysine riboswitches across all prokaryotic phyla (not counting those detected in different strains of the same species) but most of these 468 riboswitches were found upstream of lysine biosynthesis or transporter genes of Firmicutes and Gammaproteobacteria though some are also present is a few species belonging to Thermotogae, Fusobacteria, Actinobacteria and Tenericutes phyla (see S4 File) To take into account potential false negatives during the process of lysine riboswitch detection using pHMM, we took all species of Firmicutes and Gammaproteobacteria where no lysine riboswitch was detected by pHMM, and additionally scanned them by using both CMs and RiboSW [17]. Since we did not find any new lysine riboswitch, we were able to confirm the absence of lysine riboswitch in those organisms.

### Phylogenetic analysis

Phylogenetic trees were constructed for Firmicutes, Gammaproteobacteria and “Other” classes that consisted of prokaryotes not belonging to either of the first two phyla. For phylogenetic tree construction, twenty protein families were selected from the subset of *Ciccarelli et al*. [48] that was originally used to build a highly resolved tree of life. Only those proteins that were found in 140 Firmicutes, 105 Gammaproteobacteria and 31 “other” classes of bacteria were used to build the subset (see S5 File). The protein families selected for phylogenetic tree construction was also used in our previous work [46] on the comparative genomics of purine riboswitch distribution in bacteria. The protein sequences corresponding to each COG (https://www.ncbi.nlm.nih.gov/COG/) were extracted from all the species and aligned using MUSCLE [56] and the poorly aligned regions with more than 20% gaps were trimmed using trimAl [57]. The aligned sequences were concatenated to produce a super-gene alignment which was then used to build a phylogenetic tree using neighbor-joining (NJ) as well as maximum likelihood (ML) methods. The NJ and ML trees for Firmicutes were generated with the MEGA6 package [58].The evolutionary distances were computed using the JTT matrix-based method and are in the units of the number of amino acid substitutions per site. Both trees were generated for 100 bootstrap replicates. The two trees were found to be consistent with one another except for differences in some bootstrap values that do not affect our conclusions. In the Firmicute tree, although the Bacillales and Lactobacillales orders are well resolved, some of the deep branches involving the evolutionary relation between some organisms belonging to the Clostridiales order and Bacillales, Lactobacillales and Selenomonadales have low bootstrap support. Specifically, we find that organisms belonging to the order Selenomonadales and the *Bacillales*, *Brevibacillus brevis* and *Kyrpidia tusciae* cluster within the Clostridiales order. Such a phylogenetic distribution is not surprising given that Bacillales and Clostridiales are known to be polyphylectic and paraphylectic respectively [59]. Our phylogenetic tree is also consistent with other recent studies involving Firmicutes [60]. The phylogeny for the Gammaproteobacteria phylum (Fig 4) shows that all the orders belonging to the phylum are well-resolved with high bootstrap support. In the “other” tree (Fig 5), the evolutionary relation between the phyla Fusobacteria and Thermotogae are well resolved but those between the Actinobacteria, Acidobacteria, Beta and Delta-proteobacteria have low bootstrap support. We find that riboswitch containing species belonging to the latter three phyla cluster with some Actinobacterial species. The mapping of riboswitch distribution on the phylogenetic tree was visualized using iTOL v3 [61]. Filled shapes (circle for biosynthesis and square for transporter genes) indicate the presence of a riboswitch upstream to the corresponding gene. If a riboswitch is present upstream to an operon, filled circles denote all the biosynthesis genes contained in the operon and the species names are highlighted in bold and brown color. Bootstrap fractions greater than 0.5 (corresponding to a bootstrap value of 50%) are denoted near the branch points of the phylogenetic trees.

### Analysis of horizontal gene transfer

We used explicit phylogenetic methods to analyze potential horizontal riboswitch transfer from one genus to another. In this method, individual NJ and ML gene trees were constructed using the same method described earlier and compared with the species tree. To further validate the observation of HGT event detected by explicit phylogenetic methods, we used the information of HGTree database [47] and also used the NCBI smart blast with a parallel BLASTp search to find the closest matches to high-quality sequences.

### Structure-based energy minimization method for identification of lysine riboswitches

To verify riboswitches detected upstream to hypothetical proteins and proteins that have not yet been directly implicated in lysine biosynthesis or transport, we used a structure-based energy minimization technique that predicts optimal secondary structures for the potential riboswitch candidates like the folding predictions performed in [19]. Even though the predictions can sometimes be obscured by the presence of suboptimal solutions that are relatively close in energy to the optimal one, we found well-resolved optimal secondary structure solutions in several instances that allowed us to unambiguously verify the predictions of the sequence-based methods. These sequences fit the comparative analysis derived structure of the lysine riboswitch aptamer [62] when analyzed by the energy minimization method. Suboptimal solutions by energy minimization can be computed with UNAFold [63] or the Vienna RNA package [64] and these can be compared to the riboswitch structure derived by comparative analysis from works that preceded x-ray crystallography experiments [4, 62, 65]. We chose to work with UNAFold [63] for suboptimal predictions and to compare our predictions to the comparative analysis derived structure of the lysine that is depicted in Fig 1 of *Vitreschak and Gelfand* [62].

### Annotations of transporter genes

The lysine riboswitches are found in the upstream of some genes that are labeled as hypothetical proteins. Our comparative genomics approach enabled us to implicate these genes in lysine transport and thereby systematically carry out detailed annotations of those genes. We first carried out the BLASTP search of all those proteins versus all the transporter proteins in the TCDB database (Transporter Classification Database) [66] to identify homologs to known and predicted transporters in the TCDB. The parameters for considering the homologous genes were set as follow: E-value ≤ 10–5, similarity ≥ 50%, and the sequence coverage ≥ 30%. We have functionally classified the transporter genes based on its homologous gene with known function in the TCDB that had the lowest expected value, the highest similarity score, and the highest coverage. Next, the Pfam database (http://pfam.xfam.org/) [67] was searched to identify the conserved structure domains of the transporters and TMHMM (http://www.cbs.dtu.dk/services/TMHMM/) [68] search was used to analyze a number of a transmembrane domain in the transporters. Only those hypothetical proteins that possess conserved structure domains associated with transporters, as well as transmembrane domains, are labeled as putative lysine transporters.

## Supporting information

S1 File Gross phylogenomic distribution of lysine riboswitches in Firmicutes. The length of the red bars in the outer arc of the figure is correlated with the number of lysine riboswitches found in the corresponding species. This number is specified at the top of the bar.
(TIF)

S2 File Generalized model of operon formation and death and its relevance to regulation by lysine riboswitches. (PDF)

S3 File SMART-BLAST results showing the strong similarity between the lysW gene of *S*. *oneidensis* and nhaC genes of other species that belong to the same clade. (PDF)

S4 File Details of genomes where lysine riboswitches are present, the locations of the riboswitches in the genomic sequences and the corresponding gene/operon information. (XLSX)

S5 File COGs used for the construction of the phylogenetic tree. (XLSX)

## Abbreviations

COGs: Clusters of Orthologous Groups
DAP pathway: diaminopimelate pathway
HGT: horizontal gene transfer
HRT: horizontal riboswitch transfer
pHMM: profile hidden markov model

## References

[1] Winkler WC, Breaker RR (2005) Regulation of bacterial gene expression by riboswitches. *Annu Rev Microbiol* vol. 59: 487–517. 16153177 10.1146/annurev.micro.59.030804.121336

[2] Breaker RR (2011) Prospects for Riboswitch Discovery and Analysis. *Mol Cell* 43(6):867–879. doi: 10.1016/j.molcel.2011.08.024 21925376 10.1016/j.molcel.2011.08.024 PMC4140403

[3] Serganov A, Nudler E (2013) A Decade of Riboswitches. *Cell* 152(1–2):17–24. doi: 10.1016/j.cell.2012.12.024 23332744 10.1016/j.cell.2012.12.024 PMC4215550

[4] Mandal M, Breaker RR (2004) Gene regulation by riboswitches. *Nat Rev Mol Cell Biol* 5(6):451–463. doi: 10.1038/nrm1403 15173824 10.1038/nrm1403

[5] Blount KF, Breaker RR (2006) Riboswitches as antibacterial drug targets. *Nat Biotechnol* 24(12):1558–1564. doi: 10.1038/nbt1268 17160062 10.1038/nbt1268

[6] Lunse CE, Schuller A, Mayer G (2014) The promise of riboswitches as potential antibacterial drug targets. *Int J Med Microbiol* 304(1):79–92. doi: 10.1016/j.ijmm.2013.09.002 24140145 10.1016/j.ijmm.2013.09.002

[7] Sudarsan N, Cohen-Chalamish S, Nakamura S, Emilsson GM, Breaker RR (2005) Thiamine pyrophosphate riboswitches are targets for the antimicrobial compound pyrithiamine. *Chem Biol* 12(12):1325–1335. doi: 10.1016/j.chembiol.2005.10.007 16356850 10.1016/j.chembiol.2005.10.007

[8] Blount KF, Wang JX, Lim J, Sudarsan N, Breaker RR (2007) Antibacterial lysine analogs that target lysine riboswitches. *Nat Chem Biol* 3(1):44–49. doi: 10.1038/nchembio842 17143270 10.1038/nchembio842

[9] Kim JN, Blount KF, Puskarz I, Lim J, Link KH, Breaker RR (2009) Design and Antimicrobial Action of Purine Analogues That Bind Guanine Riboswitches. *ACS Chem Biol* 4(11):915–927. doi: 10.1021/cb900146k 19739679 10.1021/cb900146k PMC4140397

[10] Howe JA, Wang H, Fischmann TO, Balibar CJ, Xiao L, Galgoci AM, Malinverni JC, Mayhood T, Villafania A, Nahvi A et al (2015) Selective small-molecule inhibition of an RNA structural element. *Nature* 526(7575):672–677. doi: 10.1038/nature15542 26416753 10.1038/nature15542

[11] Wieland M, Benz A, Klauser B, Hartig JS (2009) Artificial Ribozyme Switches Containing Natural Riboswitch Aptamer Domains. *Angew Chem Int Edit* 48(15):2715–2718. 10.1002/anie.200805311 19156802

[12] Dixon N, Duncan JN, Geerlings T, Dunstan MS, McCarthy JEG, Leys D, Micklefield J (2010) Reengineering orthogonally selective riboswitches. *PROC NATL ACAD SCI USA* 107(7):2830–2835. doi: 10.1073/pnas.0911209107 20133756 10.1073/pnas.0911209107 PMC2840279

[13] Zhou LB, Zeng AP (2015) Engineering a Lysine-ON Riboswitch for Metabolic Control of Lysine Production in Corynebacterium glutamicum. *ACS Synthetic Biology* 4(12):1335–1340. doi: 10.1021/acssynbio.5b00075 26300047 10.1021/acssynbio.5b00075

[14] Zhou LB, Zeng AP (2015) Exploring Lysine Riboswitch for Metabolic Flux Control and Improvement of L-Lysine Synthesis in Corynebacterium glutamicum. *ACS Synthetic Biology* 4(6):729–734. doi: 10.1021/sb500332c 25575181 10.1021/sb500332c

[15] Bengert P, Dandekar T (2004) Riboswitch finder—a tool for identification of riboswitch RNAs. *Nucleic Acids Res* 32:W154–W159. doi: 10.1093/nar/gkh352 15215370 10.1093/nar/gkh352 PMC441490

[16] Abreu-Goodger C, Merino E (2005) RibEx: a web server for locating riboswitches and other conserved bacterial regulatory elements. *Nucleic Acids Res* 33:W690–W692. doi: 10.1093/nar/gki445 15980564 10.1093/nar/gki445 PMC1160206

[17] Chang T-H, Huang H-D, Wu L-C, Yeh C-T, Liu B-J, Horng J-T (2009) Computational identification of riboswitches based on RNA conserved functional sequences and conformations. *RNA-a Publication of the Rna Society* 15(7):1426–1430. 10.1261/rna.1623809 PMC2704089 19460868

[18] Havill JT, Bhatiya C, Johnson SM, Sheets JD, Thompson JS (2014) A new approach for detecting riboswitches in DNA sequences. *Bioinformatics* 30(21):3012–3019. doi: 10.1093/bioinformatics/btu479 25015992 10.1093/bioinformatics/btu479 PMC4609007

[19] Retwitzer MD, Kifer I, Sengupta S, Yakhini Z, Barash D (2015) An Efficient Minimum Free Energy Structure-Based Search Method for Riboswitch Identification Based on Inverse RNA Folding. *Plos One* 10(7). 10.1371/journal.pone.0134262 PMC4521916 26230932

[20] Retwitzer MD, Polishchuk M, Churkin E, Kifer I, Yakhini Z, Barash D (2015) RNAPattMatch: a web server for RNA sequence/structure motif detection based on pattern matching with flexible gaps. *Nucleic Acids Res* 43(W1):W507–W512. doi: 10.1093/nar/gkv435 25940619 10.1093/nar/gkv435 PMC4489251

[21] Nawrocki EP, Kolbe DL, Eddy SR (2009) Infernal 1.0: inference of RNA alignments. *Bioinformatics* 25(10):1335–1337. doi: 10.1093/bioinformatics/btp157 19307242 10.1093/bioinformatics/btp157 PMC2732312

[22] Nawrocki EP, Burge SW, Bateman A, Daub J, Eberhardt RY, Eddy SR, Floden EW, Gardner PP, Jones TA, Tate J et al (2015) Rfam 12.0: updates to the RNA families database. *Nucleic Acids Res* 43(D1):D130–D137. 25392425 10.1093/nar/gku1063 PMC4383904

[23] Singh P, Bandyopadhyay P, Bhattacharya S, Krishnamachari A, Sengupta S (2009) Riboswitch Detection Using Profile Hidden Markov Models. *BMC Bioinformatics* 10. 10.1186/1471-2105-10-325 PMC2770071 19814811

[24] Mukherjee S, Sengupta S (2016) Riboswitch Scanner: an efficient pHMM-based web-server to detect riboswitches in genomic sequences. *Bioinformatics* 32(5):776–778. doi: 10.1093/bioinformatics/btv640 26519506 10.1093/bioinformatics/btv640

[25] Rodionov DA, Vitreschak AG, Mironov AA, Gelfand MS (2002) Comparative genomics of thiamin biosynthesis in procaryotes—New genes and regulatory mechanisms. *J Biol Chem* 277(50):48949–48959. doi: 10.1074/jbc.M208965200 12376536 10.1074/jbc.M208965200

[26] Rodionov DA, Vitreschak AG, Mironov AA, Gelfand MS (2003) Comparative Genomics of the vitamin B-12 metabolism and regulation in prokaryotes. *J Biol Chem* 278(42):41148–41159. doi: 10.1074/jbc.M305837200 12869542 10.1074/jbc.M305837200

[27] Barrick JE, Breaker RR (2007) The distributions, mechanisms, and structures of metabolite-binding riboswitches. *Genome Biol* 8(11). 10.1186/gb-2007-8-11-r239 PMC2258182 17997835

[28] Sun EI, Leyn SA, Kazanov MD, Saier MH, Novichkov PS, Rodionov DA (2013) Comparative genomics of metabolic capacities of regulons controlled by cis-regulatory RNA motifs in bacteria. *BMC Genomics* 14. 10.1186/1471-2164-14-597 PMC3766115 24060102

[29] Breaker RR (2012) Riboswitches and the RNA World. *Cold Spring Harbor Perspectives in Biology* 4(2). 10.1101/cshperspect.a003566 PMC3281570 21106649

[30] Rodionov DA, Vitreschak AG, Mironov AA, Gelfand MS (2003) Regulation of lysine biosynthesis and transport genes in bacteria: yet another RNA riboswitch? *Nucleic Acids Res* 31(23):6748–6757. doi: 10.1093/nar/gkg900 14627808 10.1093/nar/gkg900 PMC290268

[31] Nishida H, Nishiyama M, Kobashi N, Kosuge T, Hoshino T, Yamane H (1999) A prokaryotic gene cluster involved in synthesis of lysine through the amino adipate pathway: A key to the evolution of amino acid biosynthesis. *Genome Res* 9(12):1175–1183. 10613839 10.1101/gr.9.12.1175

[32] Nishida H (2001) Distribution of genes for lysine biosynthesis through the aminoadipate pathway among prokaryotic genomes. *Bioinformatics* 17(2):189–191. 11238076 10.1093/bioinformatics/17.2.189

[33] Born TL, Blanchard JS (1999) Structure/function studies on enzymes in the diaminopimelate pathway of bacterial cell wall biosynthesis. *Curr Opin Chem Biol* 3(5):607–613. 10508663 10.1016/s1367-5931(99)00016-2

[34] Bugg TDH, Brandish PE (1994) From Peptidoglycan to Glycoproteins—ommon features of lipid-linked oligosaccharide biosynthesis. *FEMS Microbiol Lett* 119(3):255–262. 8050708 10.1111/j.1574-6968.1994.tb06898.x

[35] Flores-Kim J, Darwin AJ (2014) Regulation of bacterial virulence gene expression by cell envelope stress responses. *Virulence* 5(8):835–851. doi: 10.4161/21505594.2014.965580 25603429 10.4161/21505594.2014.965580 PMC4601401

[36] Itoh T, Takemoto K, Mori H, Gojobori T (1999) Evolutionary instability of operon structures disclosed by sequence comparisons of complete microbial genomes. *Mol Biol Evol* 16(3):332–346. 10331260 10.1093/oxfordjournals.molbev.a026114

[37] Price MN, Huang KH, Arkin AP, Alm EJ (2005) Operon formation is driven by co-regulation and not by horizontal gene transfer. *Genome Res* 15(6):809–819. doi: 10.1101/gr.3368805 15930492 10.1101/gr.3368805 PMC1142471

[38] Price MN, Arkin AP, Alm EJ (2006) The life-cycle of operons. *Plos Genet* 2(6):859–873. 10.1371/journal.pgen.0020096 PMC1480536 16789824

[39] Arellano-Reynoso B, Lapaque N, Salcedo S, Briones G, Ciocchini AE, Ugalde R, Moreno E, Moriyon I, Gorvel JP (2005) Cyclic beta-1,2-glucan is a brucella virulence factor required for intracellular survival. *Nat Immunol* 6(6):618–625. doi: 10.1038/ni1202 15880113 10.1038/ni1202

[40] Ciocchini AE, Guidolin LS, Casabuono AC, Couto AS, de Iannino NI, Ugalde RA (2007) A glycosyltransferase with a length-controlling activity as a mechanism to regulate the size of polysaccharides. *PROC NATL ACAD SCI USA* 104(42):16492–16497. doi: 10.1073/pnas.0708025104 17921247 10.1073/pnas.0708025104 PMC2034269

[41] Bastet L, Dube A, Masse E, Lafontaine DA (2011) New insights into riboswitch regulation mechanisms. *Mol Microbiol* 80(5):1148–1154. doi: 10.1111/j.1365-2958.2011.07654.x 21477128 10.1111/j.1365-2958.2011.07654.x

[42] Caron MP, Bastet L, Lussier A, Simoneau-Roy M, Masse E, Lafontaine DA (2012) Dual-acting riboswitch control of translation initiation and mRNA decay. *PROC NATL ACAD SCI USA* 109(50):E3444–E3453. doi: 10.1073/pnas.1214024109 23169642 10.1073/pnas.1214024109 PMC3528543

[43] Mellin JR, Cossart P (2015) Unexpected versatility in bacterial riboswitches. *Trends Genet* 31(3):150–156. doi: 10.1016/j.tig.2015.01.005 25708284 10.1016/j.tig.2015.01.005

[44] Serganov A, Patel DJ (2009) Amino acid recognition and gene regulation by riboswitches. *BBA-Gene Regul Mech* 1789(9–10):592–611. 10.1016/j.bbagrm.2009.07.002 PMC3744886 19619684

[45] Dam P, Olman V, Harris K, Su ZC, Xu Y (2007) Operon prediction using both genome-specific and general genomic information. *Nucleic Acids Res* 35(1):288–298. doi: 10.1093/nar/gkl1018 17170009 10.1093/nar/gkl1018 PMC1802555

[46] Singh P, Sengupta S (2012) Phylogenetic Analysis and Comparative Genomics of Purine Riboswitch Distribution in Prokaryotes. *Evol Bioinform* 8:589–609. 10.4137/EBO.S10048 PMC3499989 23170063

[47] Jeong H, Sung S, Kwon T, Seo M, Caetano-Anolles K, Choi SH, Choi S, Nasir A, Kim H (2016) HGTree: database of horizontally transferred genes determined by tree reconciliation. *Nucleic Acids Res* 44(D1):D610–D619. doi: 10.1093/nar/gkv1245 26578597 10.1093/nar/gkv1245 PMC4702880

[48] Ciccarelli FD, Doerks T, von Mering C, Creevey CJ, Snel B, Bork P (2006) Toward automatic reconstruction of a highly resolved tree of life. *Science* 311(5765):1283–1287. doi: 10.1126/science.1123061 16513982 10.1126/science.1123061

[49] Hug LA, Baker BJ, Anantharaman K, Brown CT, Probst AJ, Castelle CJ, Butterfield CN, Hernsdorf AW, Amano Y, Ise K et al (2016) A new view of the tree of life. *Nat Microbiol* 1(5). 10.1038/nmicrobiol.2016.48 27572647

[50] Munoz-Gomez SA, Roger AJ (2016) Leaving negative ancestors behind. *Elife* 5. 10.7554/eLife.20061 PMC5007113 27580371

[51] Tatusova T, Ciufo S, Fedorov B, O'Neill K, Tolstoy I (2015) RefSeq microbial genomes database: new representation and annotation strategy (vol 42, pg 553, 2014). *Nucleic Acids Res* 43(7):3872–3872. doi: 10.1093/nar/gkv278 25824943 10.1093/nar/gkv278 PMC4402550

[52] Mao FL, Dam P, Chou J, Olman V, Xu Y (2009) DOOR: a database for prokaryotic operons. *Nucleic Acids Res* 37:D459–D463. doi: 10.1093/nar/gkn757 18988623 10.1093/nar/gkn757 PMC2686520

[53] Mao XZ, Ma Q, Zhou C, Chen X, Zhang HY, Yang JC, Mao FL, Lai W, Xu Y (2014) DOOR 2.0: presenting operons and their functions through dynamic and integrated views. *Nucleic Acids Res* 42(D1):D654–D659. 24214966 10.1093/nar/gkt1048 PMC3965076

[54] Taboada B, Ciria R, Martinez-Guerrero CE, Merino E (2012) ProOpDB: Prokaryotic Operon DataBase. *Nucleic Acids Res* 40(D1):D627–D631. 22096236 10.1093/nar/gkr1020 PMC3245079

[55] Quinlan AR, Hall IM (2010) BEDTools: a flexible suite of utilities for comparing genomic features. *Bioinformatics* 26(6):841–842. doi: 10.1093/bioinformatics/btq033 20110278 10.1093/bioinformatics/btq033 PMC2832824

[56] Edgar RC (2004) MUSCLE: multiple sequence alignment with high accuracy and high throughput. *Nucleic Acids Res* 32(5):1792–1797. doi: 10.1093/nar/gkh340 15034147 10.1093/nar/gkh340 PMC390337

[57] Capella-Gutierrez S, Silla-Martinez JM, Gabaldon T (2009) trimAl: a tool for automated alignment trimming in large-scale phylogenetic analyses. *Bioinformatics* 25(15):1972–1973. doi: 10.1093/bioinformatics/btp348 19505945 10.1093/bioinformatics/btp348 PMC2712344

[58] Tamura K, Stecher G, Peterson D, Filipski A, Kumar S (2013) MEGA6: Molecular Evolutionary Genetics Analysis Version 6.0. *Mol Biol Evol* 30(12):2725–2729. doi: 10.1093/molbev/mst197 24132122 10.1093/molbev/mst197 PMC3840312

[59] Wolf M, Müller T, Dandekar T, Pollack JD (2004) Phylogeny of Firmicutes with special reference to Mycoplasma (Mollicutes) as inferred from phosphoglycerate kinase amino acid sequence data. *Int J Syst Evol Microbiol* 54(3): 871–875. 15143038 10.1099/ijs.0.02868-0

[60] Zhang WW, Lu ZT (2015) Phylogenomic evaluation of members above the species level within the phylum Firmicutes based on conserved proteins. *Environ Microbiol Reports* 7(2):273–281. 10.1111/1758-2229.12241 25403554

[61] Letunic I, Bork P (2016) Interactive tree of life (iTOL) v3: an online tool for the display and annotation of phylogenetic and other trees. *Nucleic Acids Res* 44(W1):W242–W245. doi: 10.1093/nar/gkw290 27095192 10.1093/nar/gkw290 PMC4987883

[62] Vitreschak AG, Rodionov DA, Mironov AA, Gelfand MS (2004) Riboswitches: the oldest mechanism for the regulation of gene expression? *Trends Genet* 20(1):44–50. doi: 10.1016/j.tig.2003.11.008 14698618 10.1016/j.tig.2003.11.008

[63] Markham, N.R, Zuker, M (2008) UNAFold. Bioinformatics: Structure, Function and Applications pp.3-31.

[64] Lorenz R, Bernhart SH, Siederdissen CHZ, Tafer H, Flamm C, Stadler PF, Hofacker IL (2011) ViennaRNA Package 2.0. *Algorithm Mol Biol* 6. 10.1186/1748-7188-6-26 PMC3319429 22115189

[65] Nudler E, Mironov AS (2004) The riboswitch control of bacterial metabolism. *Trends in Biochemical Sciences* 29(1):11–17. doi: 10.1016/j.tibs.2003.11.004 14729327 10.1016/j.tibs.2003.11.004

[66] Saier MH, Tran CV, Barabote RD (2006) TCDB: the Transporter Classification Database for membrane transport protein analyses and information. *Nucleic Acids Res* 34:D181–D186. doi: 10.1093/nar/gkj001 16381841 10.1093/nar/gkj001 PMC1334385

[67] Bateman A, Birney E, Durbin R, Eddy SR, Howe KL, Sonnhammer ELL (2000) The Pfam protein families database. *Nucleic Acids Res* 28(1):263–266. 10592242 10.1093/nar/28.1.263 PMC102420

[68] Krogh A, Larsson B, von Heijne G, Sonnhammer ELL (2001) Predicting transmembrane protein topology with a hidden Markov model: Application to complete genomes. *J Mol Biol* 305(3):567–580. doi: 10.1006/jmbi.2000.4315 11152613 10.1006/jmbi.2000.4315

