# Supplementary figures and images for: Comparative genomics and phylogenomic analyses of lysine riboswitch distributions in bacteria
Source: PLoS One. 2017 Sep 5;12(9):e0184314. doi: 10.1371/journal.pone.0184314 (PMC5584792; doi:10.1371/journal.pone.0184314)

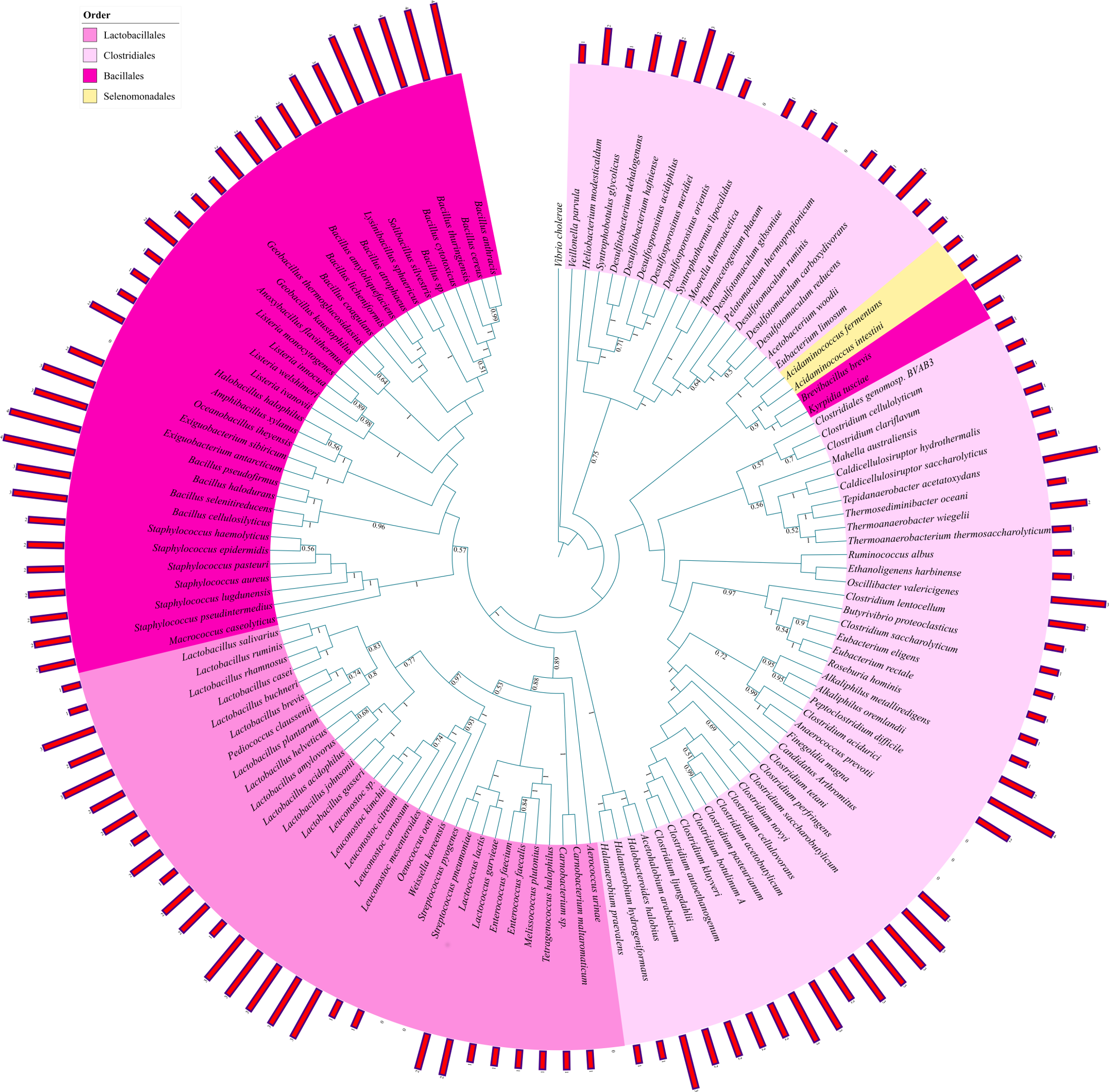

Supplement: S1 File — The length of the red bars in the outer arc of the figure is correlated with the number of lysine riboswitches found in the corresponding species. This number is specified at the top of the bar. (TIF) [file pone.0184314.s001.tif]

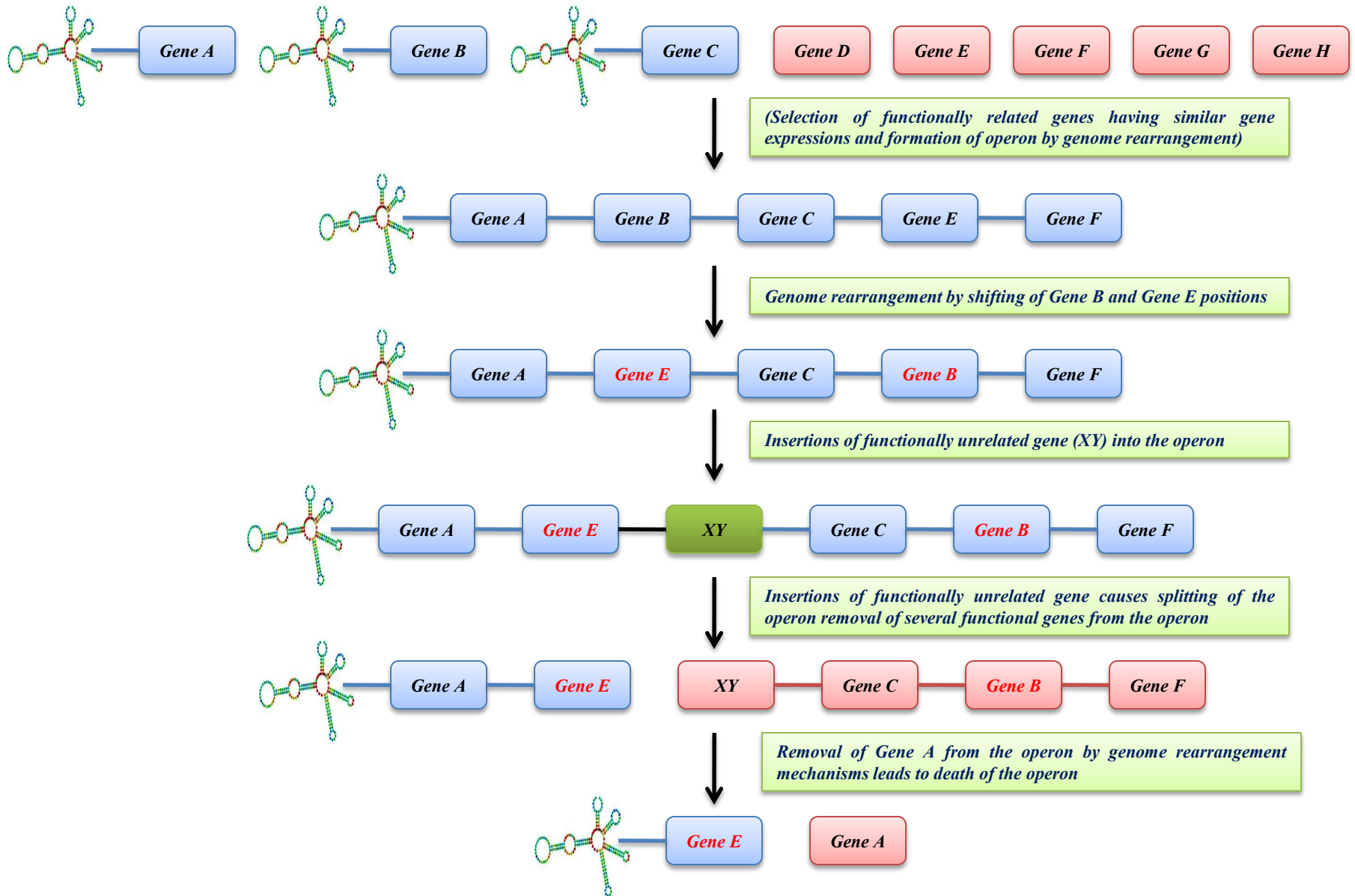

Supplement: S2 File — (PDF) [file pone.0184314.s002.pdf]
