# Supplementary material for: Comparative genomics and phylogenomic analyses of lysine riboswitch distributions in bacteria
Source: PLoS One. 2017 Sep 5;12(9):e0184314. doi: 10.1371/journal.pone.0184314 (PMC5584792; doi:10.1371/journal.pone.0184314)
Supplement: S3 File — (PDF) [file pone.0184314.s003.pdf]

SMART BLAST

Query: *Shewanella halifaxensis HAW-EB4 (nhaC-4071980-4073564)*

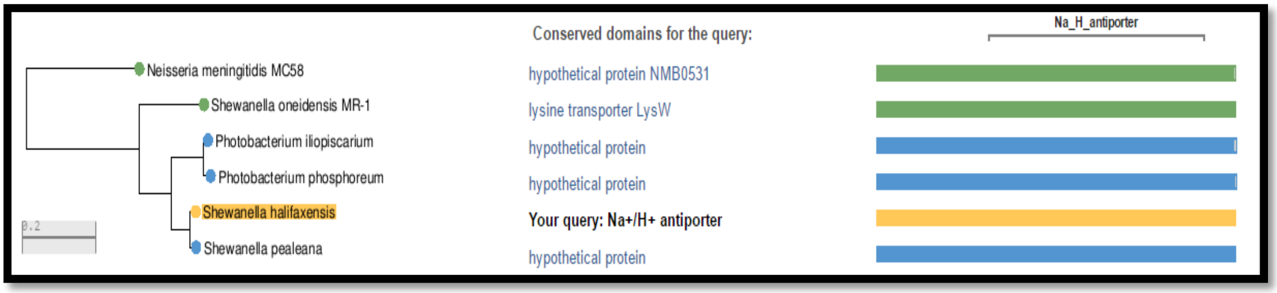

Best hits

Select: All None Selected:0

Alignments GenPept

	Description	Max score	Total score	Query cover	E value	Ident	Accession
	hypothetical protein [Shewanella pealeana]	1025	1025	100%	0.0	97%	WP_012156474.1
	hypothetical protein [Photobacterium iliopiscarium]	892	892	99%	0.0	85%	WP_045037034.1
	hypothetical protein [Photobacterium phosphoreum]	891	891	99%	0.0	84%	WP_036794242.1
	lysine transporter LysW [Shewanella oneidensis MR-1]	744	744	100%	0.0	72%	NP_716635.1
	hypothetical protein NMB0531 [Neisseria meningitidis MC58]	466	466	98%	1e-157	48%	NP_273576.1

Query: *Shewanella pealeana ATCC 700345 (nhaC-3996612-3998196)*

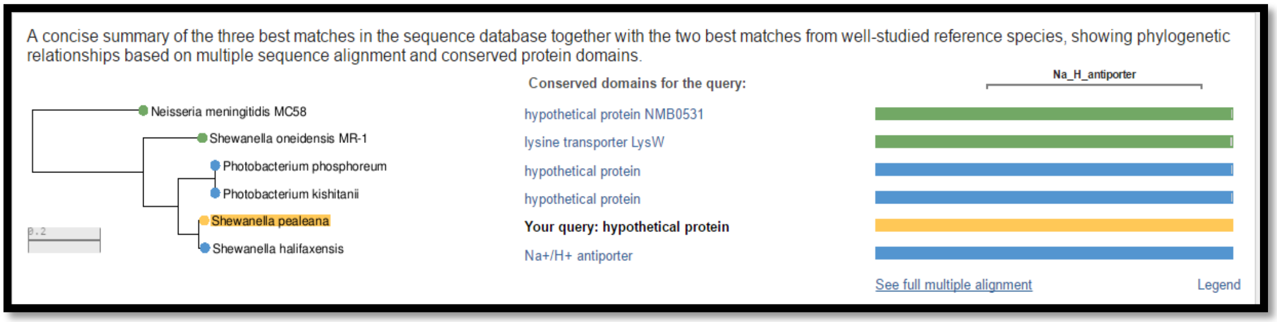

Best hits

Select: All None Selected:0

Alignments GenPept

	Description	Max score	Total score	Query cover	E value	Ident	Accession
	Na+/H+ antiporter [Shewanella halifaxensis]	1025	1025	100%	0.0	97%	WP_012278401.1
	hypothetical protein [Photobacterium phosphoreum]	895	895	99%	0.0	85%	WP_036794242.1
	hypothetical protein [Photobacterium kishitanii]	895	895	99%	0.0	85%	WP_045041850.1
	lysine transporter LysW [Shewanella oneidensis MR-1]	748	748	99%	0.0	72%	NP_716635.1
	hypothetical protein NMB0531 [Neisseria meningitidis MC58]	462	462	98%	6e-156	48%	NP_273576.1

Query: *Photobacterium profundum SS9 (nhaC-3172989-3174573)*

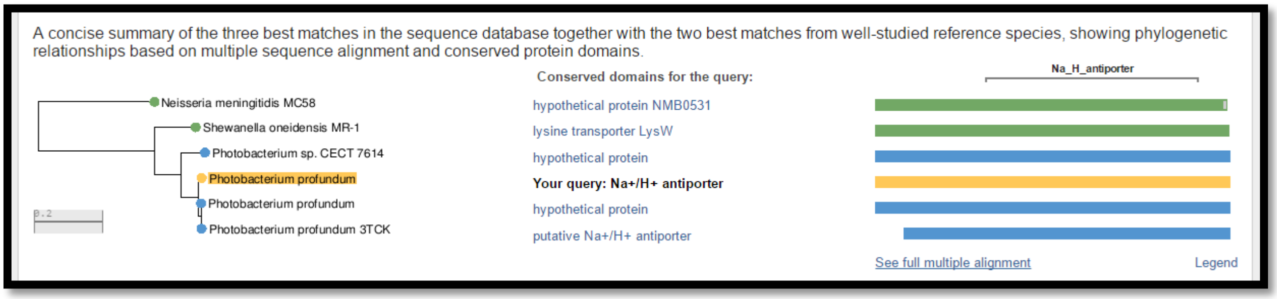

Best hits

Select: All None Selected:0

Alignments GenPept

	Description	Max score	Total score	Query cover	E value	Ident	Accession
	hypothetical protein [Photobacterium profundum]	1047	1047	100%	0.0	98%	WP_036804011.1
	putative Na+/H+ antiporter [Photobacterium profundum 3TCK]	966	966	92%	0.0	98%	EAS42662.1
	hypothetical protein [Photobacterium aphoticum]	922	922	100%	0.0	87%	WP_047873042.1
	lysine transporter LysW [Shewanella oneidensis MR-1]	815	815	99%	0.0	77%	NP_716635.1
	hypothetical protein NMB0531 [Neisseria meningitidis MC58]	454	454	98%	6e-153	47%	NP_273576.1

Query: *Aeromonas salmonicida subsp. salmonicida A449*

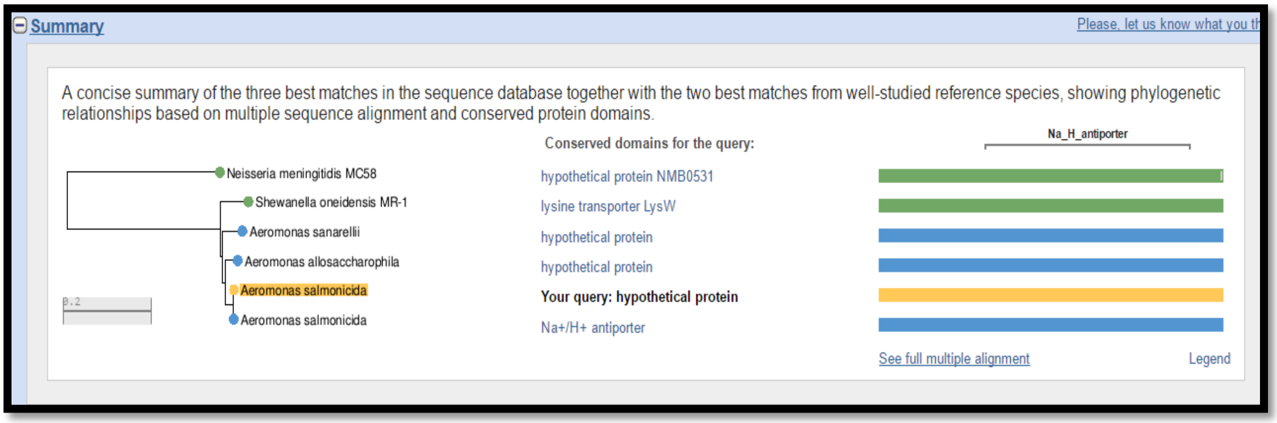

Best hits

Select: All None Selected:0

Alignments GenPept

	Description	Max score	Total score	Query cover	E value	Ident	Accession
	Na+/H+ antiporter [Aeromonas salmonicida]	1053	1053	100%	0.0	99%	WP_058394088.1
	hypothetical protein [Aeromonas allosaccharophila]	1015	1015	100%	0.0	95%	WP_042057294.1
	hypothetical protein [Aeromonas sanarellii]	991	991	100%	0.0	93%	WP_042076508.1
	lysine transporter LysW [Shewanella oneidensis MR-1]	939	939	100%	0.0	90%	NP_716635.1
	hypothetical protein NMB0531 [Neisseria meningitidis MC58]	468	468	98%	1e-158	50%	NP_273576.1

Query: *Aeromonas veronii B565*

A concise summary of the three best matches in the sequence database together with the two best matches from well-studied reference species, showing phylogenetic relationships based on multiple sequence alignment and conserved protein domains.

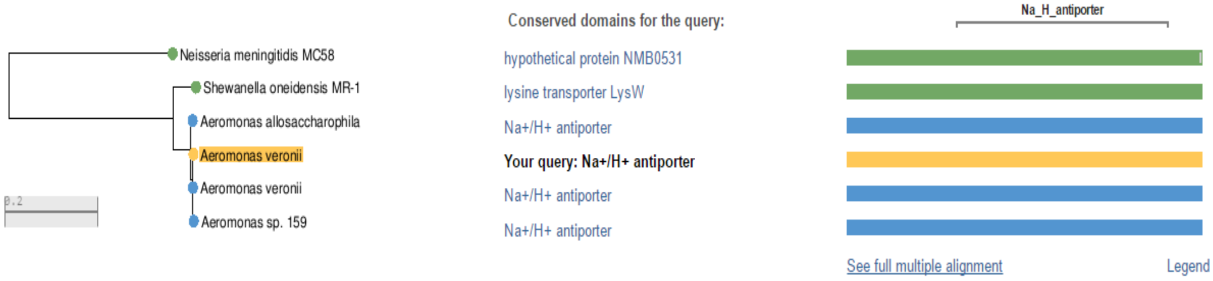

⊖ Best hits

Select: All None Selected:0

Alignments GenPept

	Description	Max score	Total score	Query cover	E value	Ident	Accession
	Na+/H+ antiporter [Aeromonas veronii]	1049	1049	100%	0.0	99%	WP_005359896.1
	Na+/H+ antiporter [Aeromonas sp. 159]	1048	1048	100%	0.0	99%	WP_019444693.1
	Na+/H+ antiporter [Aeromonas allosaccharophila]	1045	1045	100%	0.0	99%	WP_058051766.1
	lysine transporter LysW [Shewanella oneidensis MR-1]	946	946	100%	0.0	91%	NP_716635.1
	hypothetical protein NMB0531 [Neisseria meningitidis MC58]	450	450	98%	2e-151	48%	NP_273576.1

Query: *Oceanimonas sp. GK1*

A concise summary of the three best matches in the sequence database together with the two best matches from well-studied reference species, showing phylogenetic relationships based on multiple sequence alignment and conserved protein domains.

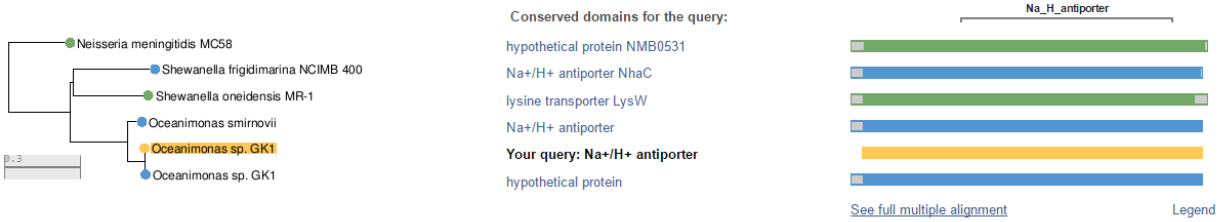

⊖ Best hits

Select: All None Selected:0

Alignments GenPept

	Description	Max score	Total score	Query cover	E value	Ident	Accession
	hypothetical protein [Oceanimonas sp. GK1]	985	985	100%	0.0	99%	WP_041543184.1
	Na+/H+ antiporter [Oceanimonas smirnovii]	875	875	100%	0.0	88%	WP_019934824.1
	Na+/H+ antiporter NhaC [Shewanella frigidimarina NCIMB 400]	543	543	99%	0.0	56%	ABI72586.1
	lysine transporter LysW [Shewanella oneidensis MR-1]	542	542	98%	0.0	58%	NP_716635.1
	hypothetical protein NMB0531 [Neisseria meningitidis MC58]	452	452	99%	1e-152	48%	NP_273576.1

Query sequence Best matches Reference species

Not aligned to query Gap
